# Wnt5a induces renal AQP2 expression by activating calcineurin signalling pathway

**DOI:** 10.1038/ncomms13636

**Published:** 2016-11-28

**Authors:** Fumiaki Ando, Eisei Sohara, Tetsuji Morimoto, Naofumi Yui, Naohiro Nomura, Eriko Kikuchi, Daiei Takahashi, Takayasu Mori, Alain Vandewalle, Tatemitsu Rai, Sei Sasaki, Yoshiaki Kondo, Shinichi Uchida

**Affiliations:** 1 Department of Nephrology, Tokyo Medical and Dental University, Tokyo 113-8510, Japan; 2 Division of Pediatrics, Tohoku Medical and Pharmaceutical University, Miyagi 983-8512, Japan; 3 Centre de Recherche sur l'Inflammation (CRI), UMRS 1149, Université Denis Diderot—Paris 7, 75018 Paris, France; 4 Department of Health Care Services Management, Nihon University School of Medicine, Tokyo 173-8610, Japan

## Abstract

Heritable nephrogenic diabetes insipidus (NDI) is characterized by defective urine concentration mechanisms in the kidney, which are mainly caused by loss-of-function mutations in the vasopressin type 2 receptor. For the treatment of heritable NDI, novel strategies that bypass the defective vasopressin type 2 receptor are required to activate the aquaporin-2 (AQP2) water channel. Here we show that Wnt5a regulates AQP2 protein expression, phosphorylation and trafficking, suggesting that Wnt5a is an endogenous ligand that can regulate AQP2 without the activation of the classic vasopressin/cAMP signalling pathway. Wnt5a successfully increases the apical membrane localization of AQP2 and urine osmolality in an NDI mouse model. We also demonstrate that calcineurin is a key regulator of Wnt5a-induced AQP2 activation without affecting intracellular cAMP level and PKA activity. The importance of calcineurin is further confirmed with its activator, arachidonic acid, which shows vasopressin-like effects underlining that calcineurin activators may be potential therapeutic targets for heritable NDI.

## Abstract (web summary)

The water channel AQP2 mediates the concentration of urine in the kidney. Here Ando *et al*. show that Wnt5 promotes collecting duct permeability by regulating AQP2 expression and localization through activation of the calmodulin/calcineurin signalling pathway.

## Graphical abstract

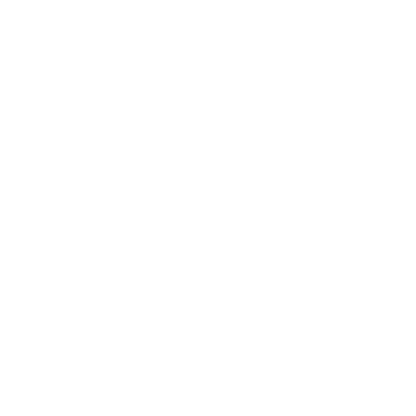

Heritable nephrogenic diabetes insipidus (NDI) is characterized by a defect in urine concentrating ability. Patients with heritable NDI are at a high risk of physical and mental retardation associated with repeated episodes of dehydration^1^. In addition, a large urine output induces non-obstructive urinary tract dilatation resulting in hydronephrotic atrophy and reduced renal function^2^. To prevent these complications, frequent drinking and urination are recommended; however, the quality of life is seriously decreased^3^.

Heritable NDI is caused by mutations in two genes. Mutations in the vasopressin type 2 receptor (V2R) account for 90% of all diagnosed heritable NDI, and mutations in the aquaporin-2 (AQP2) water channel occur in the other 10% (ref. 4). V2R and AQP2 are critical determinants of urine concentration regulation, and the vasopressin signalling pathway that regulates AQP2 trafficking has been well established. Circulating vasopressin binds to V2R and activates adenyl cyclase resulting in increased cAMP production. An elevated cAMP concentration induces phosphorylation of AQP2 at serine 256, which triggers the accumulation of AQP2 at the apical plasma membrane to increase water reabsorption. On the other hand, the presence of cAMP-independent mechanism of AQP2 regulation has been postulated^5^. For the treatment of heritable NDI caused by V2R mutations, bypassing defective V2R is required to increase AQP2 at the apical plasma membrane^6^,^7^,^8^,^9^,^10^,^11^,^12^,^13^,^14^; however, no specific pharmacological therapy is currently available.

Although precise mechanisms are still unknown, intracellular calcium mobilization affects apical trafficking of AQP2 (refs 15, 16), suggesting that calcium signalling is a major target for the treatment of heritable NDI. Therefore, we focused on classic calcium signal transducer, Wnt5a (ref. 17). Wnt5a is a ligand for frizzled (Fzd) receptors and is proved to increase intracellular calcium in several cell culture models^18^,^19^,^20^,^21^. Genetic researches reveal that Wnt5a plays an important role in the development of various organs. Wnt5a mutations cause autosomal dominant Robinow syndrome, characterized by short stature, limb shortening, genital hypoplasia and craniofacial abnormalities^22^. Wnt5a knockout mice present a phenotype of skeletal dysplasia similar to Robinow syndrome, and exhibit ventricular septal defects and respiratory dysfunction, resulting in neonatal lethality^23^. Recent reports indicate that Wnt5a is involved in not only developmental processes but also postnatal events and diseases, such as cancer, rheumatoid arthritis, obesity and insulin resistance^24^. However, little is known about the role of Wnt5a in adult kidney. In this study, we reported that Wnt5a caused apical AQP2 trafficking by the activation of calcium/calmodulin/calcineurin signalling pathway and increased urine concentration in an NDI mouse model, suggesting that Wnt5a may be a potential therapeutic target for heritable NDI.

## Results

### Wnt5a regulates AQP2 phosphorylation at S261 and S269

**Figure 1:**
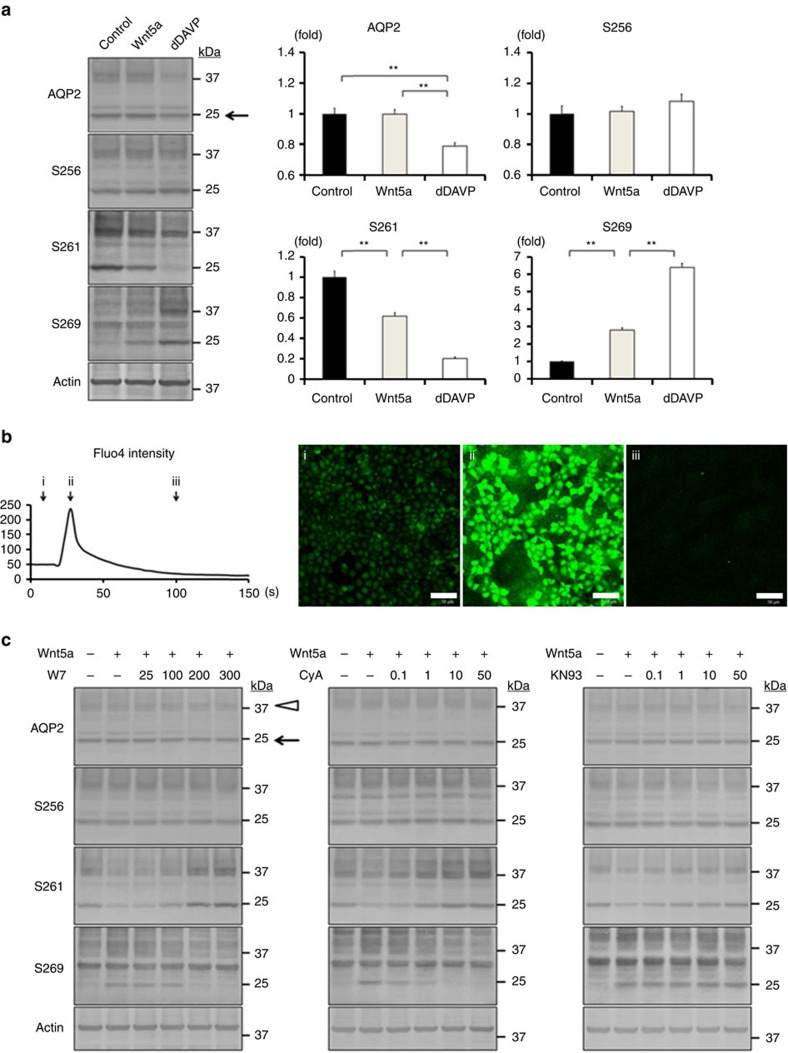
Wnt5a alters AQP2 phosphorylation through the activation of calcium signalling. (**a**) Western blot analysis of total and phosphorylated AQP2. (Left) Wnt5a (500 ng ml^−1^) or dDAVP (1 nM) was added to the basolateral side of the mpkCCD cells for 1 h. (Right) Non-glycosylated AQP2 bands (arrow) were quantified by densitometric analysis, and the results are presented in the bar graphs as fold change compared with the value in the control cells. Error bars are mean values±s.d. from three experiments. Tukey, **P<0.01. (**b**) The increase of Fluo4 intensity. (Left) Wnt5a (500 ng ml^−1^) was added to the mpkCCD cells. The time-course of Fluo4 fluorescence intensity is shown. The *x* axis indicates time, and the *y* axis indicates Fluo4 intensity. (Right) Representative confocal images at the indicated time (arrow) are shown. Scale bars, 50 μm. (**c**) Dose–response studies of W7, CyA, and KN93. Wnt5a (500 ng ml^−1^) was added in the presence or absence of W7 (25–300 μM), CyA (0.1–50 μM), or KN93 (0.1–50 μM) for 1 h. The mpkCCD cells were pretreated with each inhibitor for 45 min before Wnt5a stimulation. Representative blots of three independent experiments are shown.

The influence of Wnt5a on AQP2 was examined *in vitro* using mouse cortical collecting duct (mpkCCD_cl4_) cells, which exhibit endogenous expression of AQP2 (refs 25, 26). Although protein expression of endogenous Fzd receptors were not detected by western blot analysis with commercially available antibodies we used, we confirmed the mRNA expression of Fzd receptors by RT-PCR (Supplementary Fig. 1a,b)^17^,^27^. The mpkCCD cells were cultured on filters, and Wnt5a was administered to the basolateral side of the mpkCCD cells. We evaluated AQP2 phosphorylation at serine 256 (S256), 261 (S261) and 269 (S269) because these phosphorylation sites are responsible for AQP2 trafficking^28^,^29^,^30^. The effects of Wnt5a were compared with those of [deamino-Cys1, d-Arg8]-vasopressin (dDAVP), as positive control. Although Wnt5a did not change AQP2 phosphorylation at S256, dDAVP treatment for 1 h decreased total AQP2 protein expression and increased the ratio of phosphorylated AQP2 at S256 to total AQP2 (Fig. 1a; Supplementary Fig. 2a–d). Probably, dDAVP increased AQP2 degradation and decreased AQP2 protein expression in acute phase of dDAVP stimulation as previously described^26^. Importantly, Wnt5a significantly dephosphorylated AQP2 at S261 and significantly phosphorylated AQP2 at S269, similar to dDAVP. On the other hand, Wnt5a administered to the apical side of the mpkCCD cells did not change AQP2 phosphorylation status, indicating that Fzd receptors binding to Wnt5a ligands were mainly localized at the basolateral side of the mpkCCD cells (Supplementary Fig. 3a–c).

We then confirmed the involvement of calcium signalling in Wnt5a-induced AQP2 phosphorylation. We directly measured the intracellular calcium level by fluorescent calcium indicators, Fluo4. In the mpkCCD cells, Wnt5a significantly increased Fluo4 intensity (Fig. 1b). The waveform of Fluo4 intensity was compatible with those obtained in other mammalian cell lines^31^. Generally, intracellular calcium serves as a second messenger and activates its downstream signalling molecules. To clarify the key molecule in the regulation of AQP2, we performed inhibition test using pharmacological inhibitors. The calmodulin antagonist, W7, inhibited the effects of Wnt5a on AQP2 phosphorylation (Fig. 1c; Supplementary Fig. 4a–f). To investigate two main downstream effectors of calmodulin, we then used inhibitors of calcineurin and calmodulin-dependent protein kinase II (CaMKII). Although the calcineurin inhibitor cyclopsporin A (CyA) completely reversed the effects of Wnt5a, the CaMKII inhibitor KN93 had only a marginal effect on AQP2 phosphorylation at S261. As a negative control study, we confirmed that the same concentrations of inhibitors did not affect dDAVP-induced AQP2 phosphorylation (Supplementary Fig. 5a–c). Additionally, to exclude the possibility that CyA decreased calcineurin activity by inhibiting the increase of intracellular calcium, we examined the effect of CyA on intracellular calcium. CyA did not attenuate the increase of Fluo4 intensity in response to Wnt5a (Supplementary Fig. 6), suggesting that CyA specifically inhibited calcineurin activity, but not its upstream signalling. These results indicated that Wnt5a/calcium/calmodulin/calcineurin signalling pathway regulated AQP2 phosphorylation at S261 and S269.

### Wnt5a regulates AQP2 trafficking

**Figure 2:**
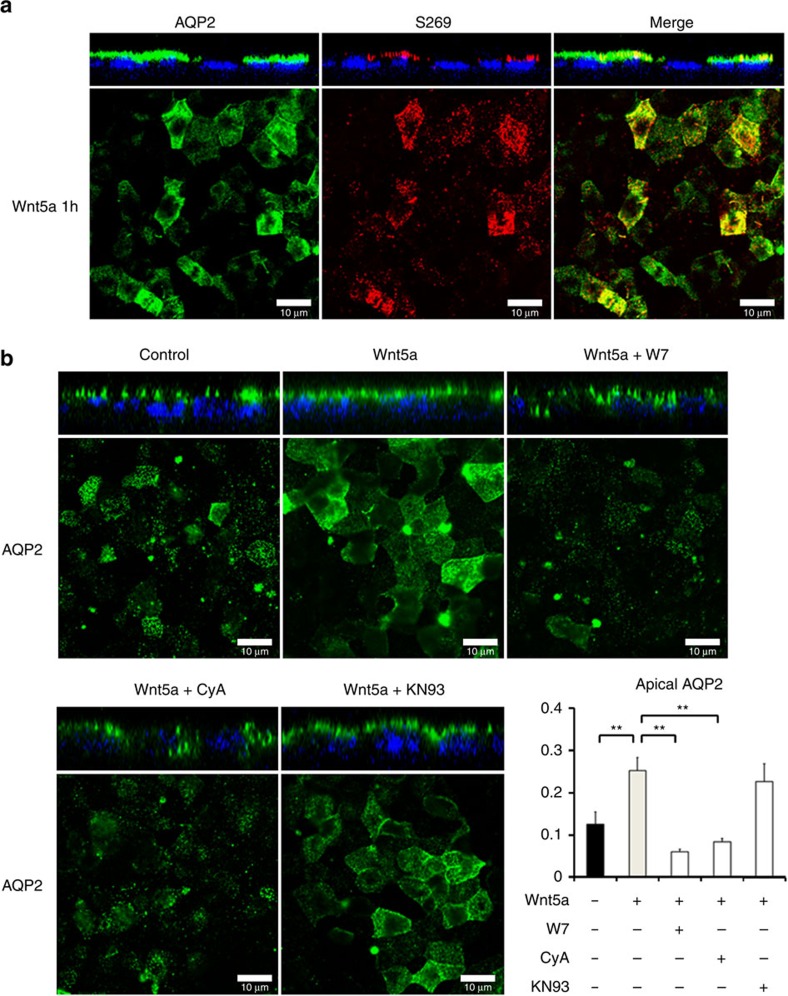
Wnt5a promotes AQP2 trafficking through the activation of calcium signalling. (**a**) The subcellular localization of total AQP2 and phosphorylated AQP2 at S269. The mpkCCD cells were treated with Wnt5a (500 ng ml^−1^) for 1 h, and the subcellular localization of AQP2 was then analysed by immunofluorescence using confocal microscopy. The larger panels display confocal sections of the apical regions of the cells. Z-stack confocal images are shown at the top of each panel. Representative confocal images of three independent experiments are shown. Scale bars, 10 μm. (**b**) The effects of W7, CyA and KN93 on Wnt5a-induced AQP2 trafficking. Wnt5a (500 ng ml^−1^) was added in the presence or absence of W7 (100 μM), CyA (10 μM), or KN93 (10 μM) for 1 h. The mpkCCD cells were pretreated with each inhibitor for 45 min before Wnt5a stimulation. Immunofluorescence staining of AQP2 was analysed as in **a**. Scale bars, 10 μm. Fluorescence intensities of apical AQP2 were quantified, and the results are presented in the bar graphs. Error bars are mean values±s.d. from three experiments. Tukey, **P<0.01.

Because phosphorylated AQP2 at S269 exclusively localizes in the apical plasma membrane of the renal collecting ducts^30^,^32^, we examined the distribution of AQP2 in the mpkCCD cells using immunofluorescent staining and confocal microscopy. Z-stack view demonstrated that total AQP2 and phosphorylated AQP2 at S269 specifically accumulated at the apical plasma membrane after Wnt5a treatment (Fig. 2a). Apical translocation of AQP2 was inhibited by W7 and CyA, but not KN93 (Fig. 2b). These results indicated that the activation of calcium signalling pathway by Wnt5a induced AQP2 trafficking as well as AQP2 phosphorylation.

### Wnt5a regulates AQP2 protein expression

**Figure 3:**
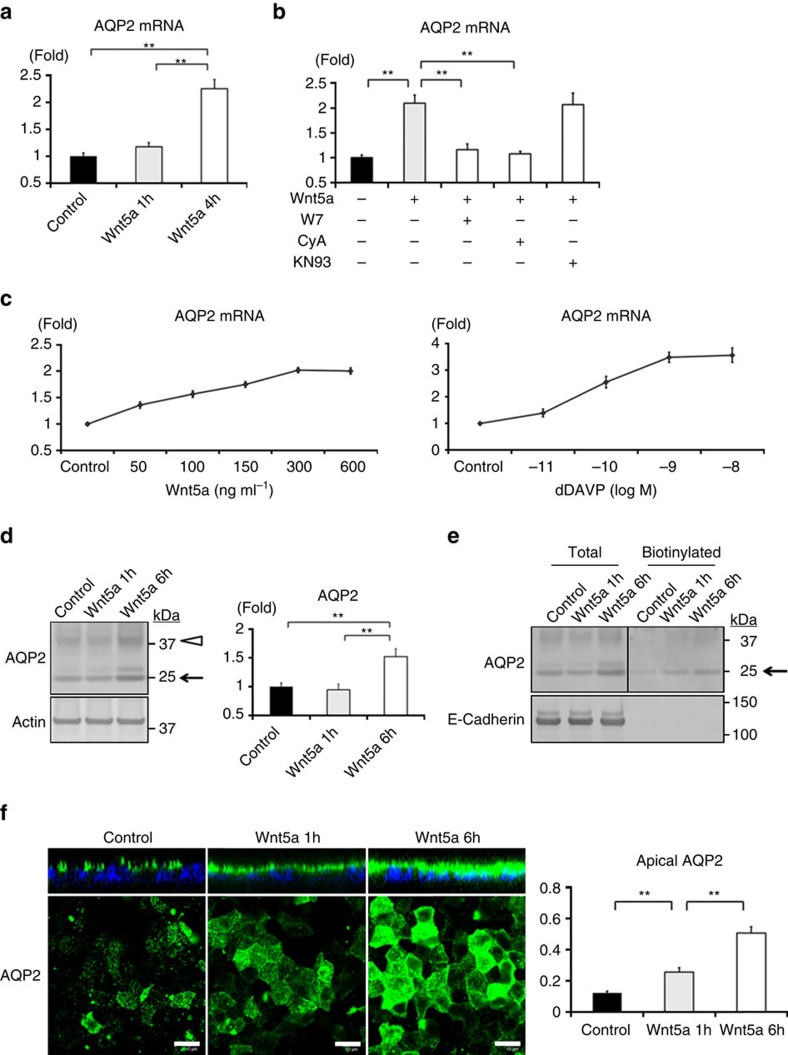
Wnt5a increases AQP2 mRNA and protein expression. (**a**) Wnt5a-induced AQP2 mRNA expression. The mpkCCD cells were treated with Wnt5a (500 ng ml^−1^) for 1 or 4 h. AQP2 mRNA expression was examined by quantitative real-time PCR. Results are presented as fold change compared with the value in the control cells. Error bars are mean values±s.d. from three experiments. Tukey, **P<0.01. (**b**) Inhibition of Wnt5a-induced AQP2 mRNA expression by W7 and CyA. Wnt5a (500 ng ml^−1^) was added to the mpkCCD cells in the presence or absence of W7 (50 μM), CyA (10 μM), or KN93 (10 μM) for 4 h. The mpkCCD cells were pretreated with each inhibitor for 45 min before Wnt5a stimulation. AQP2 mRNA expression was analyzed as in **a**. Bars are mean values±s.d. from four experiments. Tukey, **P<0.01. (**c**) Dose–response curves of AQP2 mRNA expression in response to Wnt5a or dDAVP. The mpkCCD cells were treated with the indicated concentrations of Wnt5a or dDAVP for 4 h. AQP2 mRNA expression was analyzed as in **a**. The *x* axis indicates the concentration of the Wnt5a or dDAVP, and the *y* axis indicates the relative fold change of the AQP2 mRNA. Each value is presented as mean±s.d. from three experiments. (**d**) Wnt5a-induced AQP2 protein expression. (Left) The mpkCCD cells were treated with Wnt5a (500 ng ml^−1^) for 1 or 6 h. (Right) Densitometric analysis of non-glycosylated AQP2 bands (arrow) are presented in the bar graphs as fold change compared with the value in the control cells. Error bars are mean values±s.d. from three experiments. Tukey, **P<0.01. (**e**) Biotinylation analysis of Wnt5a-induced apical AQP2 expression. The mpkCCD cells were treated with Wnt5a (500 ng ml^−1^) for 1 or 6 h. The amount of AQP2 in the apical plasma membrane was quantitated by apical surface biotinylation. Representative blots of three independent experiments are shown. (**f**) Immunofluorescent analysis of Wnt5a-induced apical AQP2 expression. (Left) The mpkCCD cells were treated with Wnt5a (500 ng ml^−1^) for 1 or 6 h. Immunofluorescence staining of AQP2 was analyzed as in Fig. 2. Scale bars, 10 μm. (Right) Fluorescence intensities of apical AQP2 were quantified, and the results are presented in the bar graphs. Error bars are mean values±s.d. from three experiments. Tukey, **P<0.01.

In addition to AQP2 trafficking, upregulation of AQP2 protein expression also contributes to the increase of apical AQP2 expression. We first assessed the effects of Wnt5a on AQP2 mRNA expression because the calcineurin signalling pathway increases AQP2 promoter activity^33^. As expected, quantitative real-time PCR analysis revealed that AQP2 mRNA increased in response to Wnt5a (Fig. 3a). W7 and CyA, but not KN93, completely inhibited the Wnt5a-induced upregulation of AQP2 mRNA expression (Fig. 3b), indicating that calcineurin activated by Wnt5a had a significant role in not only AQP2 trafficking but also AQP2 mRNA expression. In a dose–response curve analysis, Wnt5a increased AQP2 mRNA two-fold, comparable with more than half (57%) of the effect of dDAVP (Fig. 3c).

Based on these results, we subsequently examined the influence of Wnt5a on AQP2 protein expression by western blot analysis. After Wnt5a stimulation, AQP2 protein expression remained unchanged during the first hour, but markedly increased at 6 h (Fig. 3d; Supplementary Fig. 7a). We further examined the relationship between increased AQP2 protein expression and apical AQP2 expression. In apical surface biotinylation analysis, Wnt5a increased apical AQP2 expression in proportion to total AQP2 expression (Fig. 3e; Supplementary Fig. 7b). Also in immunofluorescent analysis, long-term Wnt5a stimulation increased apical AQP2 expression (Fig. 3f). These data clearly showed that Wnt5a activated AQP2, similar to vasopressin.

### Wnt5a regulates AQP2 without the activation of cAMP

**Figure 4:**
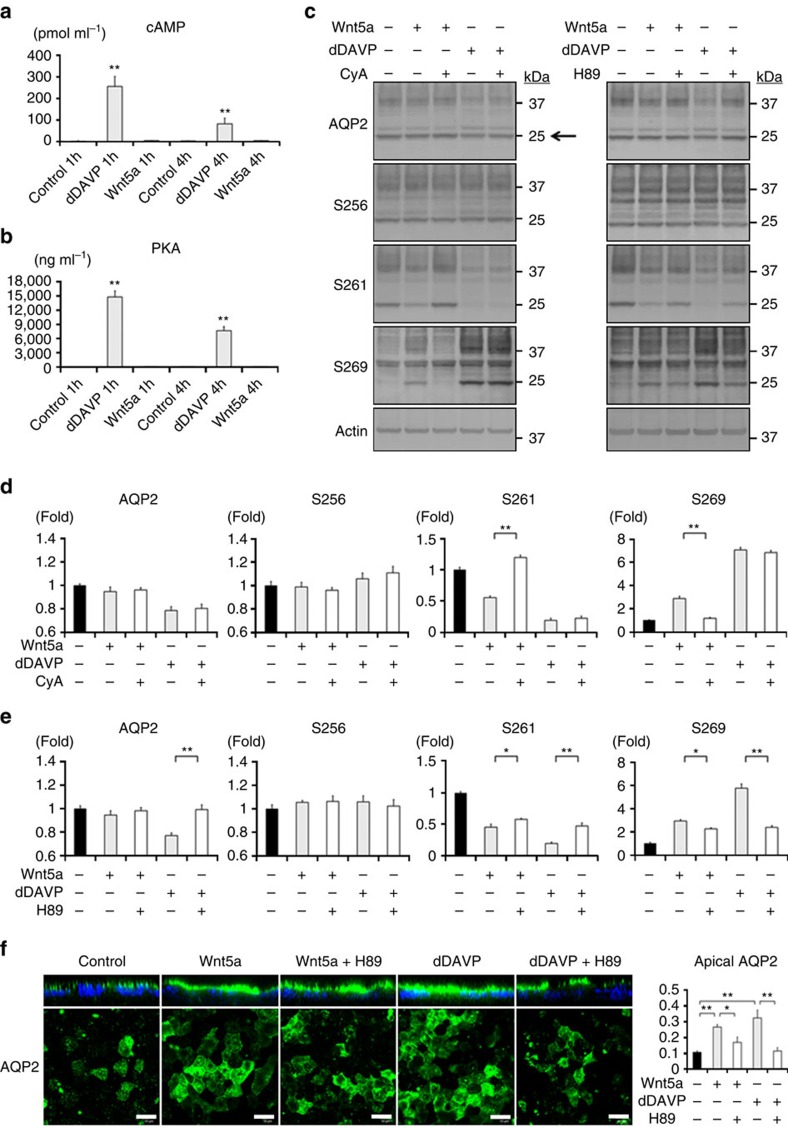
Wnt5a does not activate cAMP and PKA. (**a**) No significant elevation of cAMP concentration in response to Wnt5a. The mpkCCD cells were treated with Wnt5a (500 ng ml^−1^) or dDAVP (1 nM) for 1 or 4 h. Error bars are mean values±s.d. from three experiments. Asterisk indicates a significant difference compared with control. Tukey, **P<0.01. (**b**) No significant elevation of PKA kinase activity in response to Wnt5a. The mpkCCD cells were treated with Wnt5a (500 ng ml^−1^) or dDAVP (1 nM) for 1 or 4 h. Error bars are mean values±s.d. from three experiments. Asterisk indicates a significant difference compared with control. Tukey, **P<0.01. (**c**–**e**) The effects of CyA and H89 on AQP2 phosphorylation. Wnt5a (500 ng ml^−1^) or dDAVP (1 nM) was added in the presence or absence of CyA (10 μM) or H89 (50 μM) for 1 h. The mpkCCD cells were pretreated with each inhibitor for 45 min before Wnt5a or dDAVP stimulation. Non-glycosylated AQP2 bands (arrow) were quantified by densitometric analysis, and the results are presented in the bar graphs as fold change compared with the value in the control cells. Mean values±s.d. were determined from three experiments. Tukey, *P<0.05, **P<0.01. (**f**) The effects of CyA and H89 on AQP2 trafficking. (Left) Wnt5a (500 ng ml^−1^) or dDAVP (1 nM) was added in the presence or absence of CyA (10 μM), or H89 (50 μM) for 1 h. The mpkCCD cells were pretreated with each inhibitor for 45 min before Wnt5a or dDAVP stimulation. Immunofluorescence staining of AQP2 was analyzed as in Fig. 2. Scale bars, 10 μm. (Right) Fluorescence intensities of apical AQP2 were quantified, and the results are presented in the bar graphs. Error bars are mean values±s.d. from three experiments. Tukey, *P<0.05, **P<0.01.

The activity of Wnt5a was mainly dependent on calcium signalling pathway. We investigated whether vasopressin signalling pathway supportively interacted with the Wnt5a-mediated AQP2 regulation. In vasopressin signalling pathway, cAMP is a major second messenger, and cAMP-dependent protein kinase, PKA, is believed to phosphorylate AQP2 at S256 along with other basophilic kinases^34^,^35^. In the cAMP and PKA kinase assay, dDAVP had an robust effect on cAMP/PKA activity as expected, but Wnt5a did not elevate cAMP/PKA activity either at 1 h or 4 h after stimulation (Fig. 4a,b), suggesting that Wnt5a-regulated AQP2 phosphorylation (Fig. 1a), trafficking (Fig. 2a) and mRNA expression (Fig. 3a) could be caused by the signal different from dDAVP. The lack of change in S256/total AQP2 ratio by Wnt5a (Supplementary Fig. 2b,c) also supports this notion.

We then examined interactions between Wnt5a and vasopressin signalling pathway, using inhibitors of their downstream molecules. Figure 4c showed the effects of CyA and PKA inhibitor, H89, on AQP2 phosphorylation. CyA inhibited the effects of Wnt5a, but not dDAVP (Fig. 4d). Conversely, the effects of dDVAP were significantly inhibited by H89; however, slight inhibitory effects of H89 on Wnt5a were also observed (Fig. 4e). Consistent with these results, in immunofluorescent analysis H89 significantly inhibited the effect of dDAVP, but only slightly that of Wnt5a, on AQP2 trafficking (Fig. 4f). It was suggested that Wnt5a signalling pathway had some connections to PKA, which might partially contribute to AQP2 activation, even though PKA kinase activity measured by ELISA (Fig. 4b) and western blot analysis using a phospho-PKA substrate antibody (Supplementary Fig. 8) remained unresponsive to Wnt5a.

### Wnt5a does not affect Dishevelled, β-catenin and Rho kinase

Next, we examined the effects of Wnt5a on Wnt signalling pathway in the mpkCCD cells. The Wnt signalling network is classified into β-catenin-dependent pathway, calcium pathway and planar cell polarity pathway^24^. Typically, the phosphorylated Dishevelled (Dvl) is an essential transducer molecule for all three pathways; however, it is not absolutely required for calcium pathway^36^. In the mpkCCD cells, Wnt5a activated calcium signalling without change of Dvl2 and Dvl3 phosphorylation status (Supplementary Fig. 9a).

Among three pathways, β-catenin-dependent pathway has been reported to be involved in AQP2 regulation. In mpkCCD cells, knockdown of β-catenin decreases dDAVP-induced AQP2 protein expression^37^. In addition, phosphorylation of β-catenin at S675 by cAMP-dependent kinase activates β-catenin^38^. We examined β-catenin protein expression and phosphorylation at S675 in the mpkCCD cells after the treatment with Wnt5a or dDAVP for 1 h. Only dDAVP, but not Wnt5a, slightly increased total and phosphorylated β-catenin (Supplementary Fig. 9a). In the mpkCCD cells, Wnt5a did not affect β-catenin-dependent pathway apparently.

The planar cell polarity pathway plays a major role in cell polarization. Previous reports showed that Wnt5a promotes apical membrane polarization by regulating Rac and Rho activities, leading to translocation of apical marker, such as F-actin, to apical region of MDCK or IEC6 cells^39^,^40^. To examine whether apical membrane polarization affected AQP2 trafficking, we co-stained the mpkCCD cells with AQP2 and F-actin after the treatment with Wnt5a. Wnt5a induced apical AQP2 trafficking, but the distribution of F-actin was not changed (Supplementary Fig. 9b). We also examined the effects of Rho kinase on AQP2 phosphorylation using a Rho kinase inhibitor, Y27632, because Rho kinase is involved in membrane polarization, and S269-AQP2 lies within a Rho kinase phosphorylation motif, RXS/T. Although 10 μM Y27632 was a concentration enough to phosphorylate AQP2 at S261 in the basal condition, Y27632 did not inhibit the effect of Wnt5a on AQP2 phosphorylation at S269 (Supplementary Fig. 9c,d). These results suggested that AQP2 trafficking in response to Wnt5a was not induced by polarity changes of the mpkCCD cells.

### Wnt5a increases urine osmolality in an NDI mouse model

**Figure 5:**
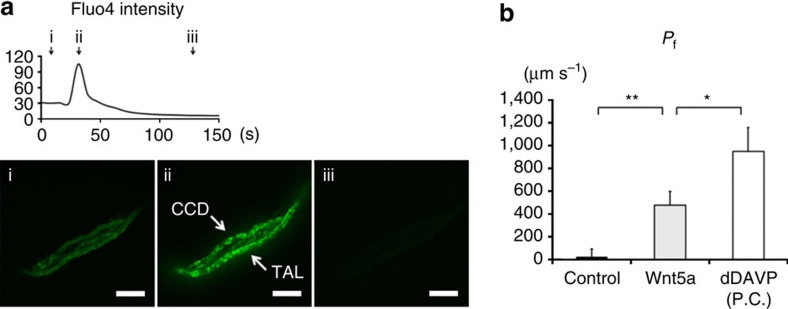
Wnt5a increases intracellular calcium and osmotic water permeability in isolated CCD of mouse kidneys. (**a**) The increase of Fluo4 intensity. (Upper) Wnt5a (500 ng ml^−1^) was added to the CCD cells dissected from mouse kidneys. The time-course of Fluo4 fluorescence intensity is shown. The *x* axis indicates time, and the *y* axis indicates Fluo4 intensity. (Lower) Representative confocal images at the indicated time (black arrow) are shown. Scale bars, 50 μm. TAL indicates thick ascending limb. (**b**) *P*_f_ in the mouse CCD. Wnt5a (500 ng ml^−1^) were added to isolated CCD tubules for 1 h. After Wnt5a washout, dDAVP (1 nM) was added to CCD for 15 min. Each value is an average of triplicate assays. P.C. indicates positive control. Mean values±s.e. were determined from six experiments. Student's *t*-test, *P<0.05, **P<0.01.

Considering that Wnt5a was found to regulate AQP2 by different mechanisms of the vasopressin signalling pathway, Wnt5a is an emerging therapeutic target for heritable NDI caused by V2R mutations. We examined the therapeutic effects of Wnt5a in an NDI mouse model. First of all, we investigated whether Wnt5a activated calcium signalling in CCD cells of mouse kidneys. We dissected renal tubules from mouse kidneys and measured the intracellular calcium levels by Fluo4. Wnt5a significantly increased Fluo4 intensity of CCD and other tubular epithelial cells, such as thick ascending limb (Fig. 5a). It was suggested that Fzd receptors were expressed in CCD cells of mouse kidneys as well as the mpkCCD cells^41^.

We then examined a functional analysis of osmotic water permeability (*P*_f_) in isolated CCD tubules using a microperfusion technique. After measurement of basal *P*_f_, Wnt5a was added to CCD. Wnt5a significantly increased *P*_f_ over the control values (Fig. 5b). To clarify maximum *P*_f_ levels of mouse CCD tubules, in each experiment, high-dose dDAVP (1 nM) was added after Wnt5a washout as positive control. The effect of Wnt5a on *P*_f_ was 54.2±11.7%, compared with that of dDAVP. These data indicated that Wnt5a actually increased osmotic water transport.

**Figure 6:**
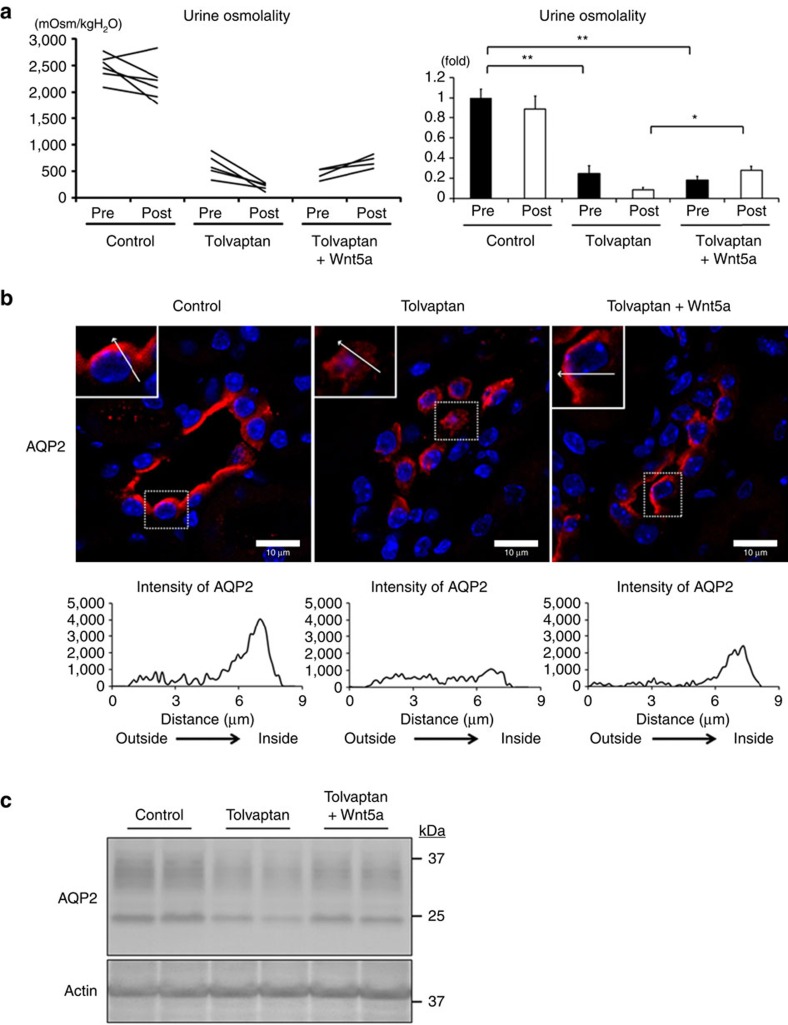
Wnt5a increases urine osmolality and apical AQP2 expression in an NDI mouse model. (**a**) Increase in urine osmolality by Wnt5a in the tolvaptan-infused mice. (Left) The C57BL/6 mice were subcutaneously infused with tolvaptan (1.5 mg h^−1^ kg^−1^) or DMSO control for 2 days by osmotic minipumps. Tolvaptan-infused mice were intraperitoneally injected with Wnt5a (500 μg kg^−1^) or PBS, and DMSO-infused control mice were intraperitoneally injected with PBS. After the injection of Wnt5a or PBS, urine was collected within 2 h. (Right) The results are presented in the bar graphs as fold change compared with the value in the control. Tukey, *P<0.05, **P<0.01. (**b**) Immunofluorescence staining of AQP2 in tolvaptan-infused mouse kidneys. (Upper) The C57BL/6 mice were subcutaneously infused with tolvaptan or DMSO control as in **a**. Tolvaptan-infused mice were intraperitoneally injected with Wnt5a (500 μg kg^−1^) or PBS, and DMSO-infused control mice were intraperitoneally injected with PBS for 1 h. Representative collecting duct cells are enlarged in the inset at top left. Scale bars, 10 μm. (Lower) The relative intensities of AQP2 staining from outer to apical membrane (along the arrow) are shown. (**c**) Western blot analysis of the membrane fraction of AQP2 in mouse kidneys. The C57BL/6 mice were subcutaneously infused with tolvaptan or DMSO control as in **a**, and then intraperitoneally injected with Wnt5a or PBS as in **b**.

Next, we examined the effects of Wnt5a *in vivo*. To generate an NDI mouse model, C57BL/6 mice were subcutaneously infused with tolvaptan or DMSO control for 2 days by osmotic minipumps^42^. After the confirmation of decreased urine osmolality by tolvaptan, Wnt5a or PBS control was intraperitoneally injected. Tolvaptan significantly decreased urine osmolality, and Wnt5a attenuated the effects of tolvaptan (Fig. 6a).

We further investigated the amounts of apical AQP2 expression. The immunofluorescent analysis of renal collecting ducts showed that AQP2 accumulated at the apical membrane in control and tolvaptan plus Wnt5a-treated mice, in contrast to the diffuse distribution of AQP2 in tolvaptan-treated mice (Fig. 6b). Similarly, the western blot analysis of the membrane fraction showed that the down-regulation of apical AQP2 in response to tolvaptan was attenuated by Wnt5a (Fig. 6c). However, the phosphorylation of AQP2 at S269 was not measurable because intraperitoneal injection of high-dose dDAVP (2.0 μg kg^−1^) was necessary to detect pS269-AQP2 bands by the antibody we used (Supplementary Fig. 10).

### Another calcineurin activator, arachidonic acid, regulates AQP2

Wnt5a is a promising therapeutic target for heritable NDI. The efficacy of Wn5a was dependent on the Wnt5a/calcium/calmodulin/calcineurin signalling pathway because Wnt5a-mediated AQP2 phosphorylation, trafficking and mRNA expression were inhibited by W7 and CyA (Figs 1c, 2b and 3b). The development of calcineurin activators is a potential therapeutic strategy for NDI treatment. We therefore investigated whether other calcineurin activators had apical-orienting effects on AQP2. Because unsaturated long-chain fatty acids, such as arachidonic acid (AA), activate calcineurin by mimicking calmodulin^43^, we analysed the effects of AA on AQP2 in the mpkCCD cells. AA altered the AQP2 phosphorylation status at S261 and S269 similar to Wnt5a, and these changes were inhibited by CyA (Supplementary Fig. 11a). In immunofluorescent analysis, AA increased apical AQP2 expression, and CyA inhibited the effect of AA (Supplementary Fig. 11b). In addition, a dose-response curve analysis showed that AA increased AQP2 mRNA expression to the same extent as that of Wnt5a (Supplementary Fig. 11c). The effect of AA on AQP2 mRNA expression was also inhibited by CyA (Supplementary Fig. 11d). Although one of the derivatives of the AA cascade, prostaglandin E2, is involved in AQP2 regulation^44^, the efficacy of AA was not attenuated by the administration of indomethacin, which inhibits the production of prostaglandins (Supplementary Fig. 12a–c). These results indicated that calcineurin activators were potential therapeutic targets for heritable NDI.

## Discussion

**Figure 7:**
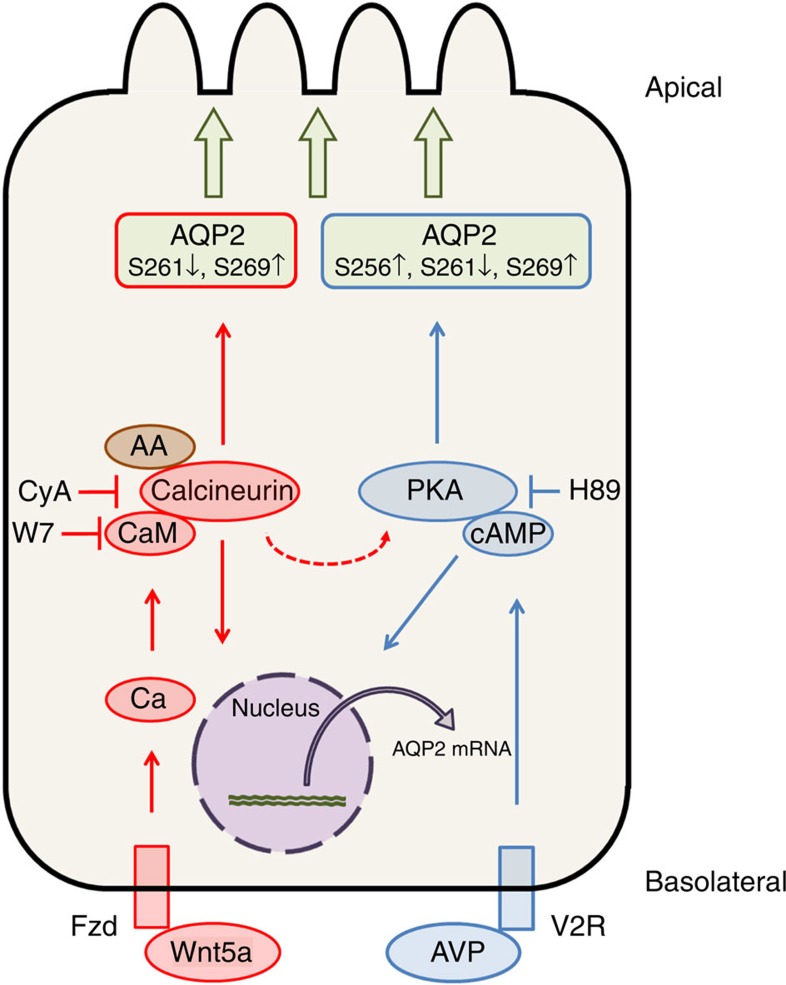
Schematic summary of Wnt5a signalling pathway in the regulation of AQP2. Wnt5a activates intracellular calcium in the presence of Fzd receptors^19^. Calcium-binding protein calmodulin (CaM) and calmodulin-mimicking protein AA stimulate calcineurin. Calcineurin alters AQP2 phosphorylation at S261 and S269 leading to the apical membrane trafficking of AQP2. Additionally, calcineurin increases AQP2 mRNA expression. On the other hand, vasopressin (AVP) binding to V2R increases intracellular cAMP concentration and activates PKA. cAMP upregulates AQP2 mRNA expression, and PKA along with other basophilic kinases alters AQP2 phosphorylation at S256, S261, and S269. Wnt5a activates AQP2 by different mechanisms of the vasopressin signalling pathway. Slight inhibitory effects of H89 on Wnt5a were also observed (Fig. 4e), suggesting the existence of crosstalk between Wnt5a signalling pathway and PKA.

In the present study, we clarified that Wnt5a regulated AQP2 phosphorylation, trafficking and protein expression (Figs 1a, 2a, 3d and 7). Wnt5a is one of the first endogenous ligands to be discovered that can regulate AQP2 without the activation of the vasopressin/cAMP signalling pathway. Instead of cAMP, Wnt5a activated calcium, calmodulin and calcineurin. Wnt5a/calcium/calmodulin/calcineurin is a novel signalling pathway in the regulation of AQP2. The importance of calcineurin was further confirmed by the fact that the calmodulin-mimicking protein, AA, also regulated AQP2 through the activation of calcineurin.

The serine/threonine phosphatase calcineurin has been reported to regulate AQP2. Calcineurin and AQP2 are co-localized at intracellular vesicles in collecting duct cells^45^. In functional analysis, calcineurin regulates AQP2 trafficking and mRNA expression^33^,^46^. Moreover, α isoform of calcineurin A subunit (CnAα) knockout mice or CyA-treated mice manifest impaired AQP2 phosphorylation at S256 and decreased urine-concentrating response to dDAVP^47^. In this study, we confirmed that calcineurin played a critical role for AQP2; however, contrary to the previous *in vivo* experiments, Wnt5a treatment did not affect AQP2 phosphorylation at S256, and CyA did not either (Figs 1a,c and 4c). In addition, CyA did not affect vasopressin-induced AQP2 phosphorylation at S256 (Fig. 4c; Supplementary Fig. 5). Probably, multiple functions of calcineurin were responsible for the different results between *in vitro* and *in vivo* studies. CnAα knockout mice simultaneously suffer from developmental abnormalities and kidney failure, which may modulate the effects of calcineurin on collecting duct cells.

Although Wnt5a did not phosphorylate AQP2 at S256, Wnt5a successfully induced AQP2 trafficking (Figs 2 and 6b). Instead of S256, Wnt5a significantly phosphorylated AQP2 at S269 (Fig. 1a). As with S256, S269 also contributes to AQP2 trafficking resulting from reduction of the AQP2 endocytosis rates^48^. Inhibition of endocytosis causes S256-independent translocation of AQP2 to the plasma membrane^49^, suggesting that the increased apical AQP2 expression by Wnt5a could be mediated mainly by the phosphorylation at S269, but not S256.

In addition to the analysis of the mpkCCD cells and NDI mouse model, we also examined the effects of Wnt5a on AQP2 using isolated CCD microperfusion experiments. Although *P*_f_ values of mouse CCD has not been reported, the increase in *P*_f_ (949±209 μm s^−1^) after 1 nM of dDAVP administration was nearly equivalent to the previous rat CCD data. In rat CCD microperfusion analysis, *P*_f_ was increased to 800±141 μm s^−1^ after 220 pM of dDAVP administration^50^. The results from dDAVP administration indicated that we successfully measured *P*_f_ of mouse CCD. Wnt5a significantly increased *P*_f_ (478±120 μm s^−1^), compared with control values (Fig. 5b). We directly confirmed that Wnt5a contributed to transcellular water transport across the tubular epithelial cells of native mouse kidneys.

The fact that the Wnt5a signalling pathway activated AQP2 provided further insights in the physiological function of Wnt5a and calcineurin in the kidney. The circulating levels of Wnt5a are increased in patients with severe sepsis and obesity^51^,^52^. Wnt5a may upregulate water reabsorption to compensate for plasma volume depletion in sepsis and may contribute to water retention in obese and metabolic syndrome patients^53^. Although calcineurin activity in the kidney has not been elucidated, its activity in the brain is significantly elevated after treatment with antipsychotics^54^,^55^. Antipsychotics are a major cause of the syndrome of inappropriate secretion of antidiuretic hormone (SIADH). The activation of calcineurin and enhancement of ADH release by antipsychotics may synergistically contribute to AQP2 activation, resulting in drug-induced SIADH.

In conclusion, we identified Wnt5a as a novel therapeutic target of heritable NDI. Wnt5a successfully increased apical AQP2 expression and urine osmolality in the V2R-inhibited NDI mouse model. In Wnt5a signalling pathway, calcineurin was a key regulator of AQP2. It was noteworthy that another calcineurin activator, AA, also had vasopressin-like effects. Screening for calcineurin activators like Wnt5a is a potential therapeutic strategy to develop novel drugs for the treatment of heritable NDI.

## Methods

### Cell culture

We generated the collecting duct cell line, mpkCCD_cl4_, as previously described^25^. The mpkCCD cells were cultured in modified DM medium (DMEM: Ham's F_12_, 1:1 vol/vol; 60 nM sodium selenate; 5 μg ml^−1^ transferrin; 2 mM glutamine; 50 nM dexamethasone; 1 nM triiodothyronine; 10 ng ml^−1^ epidermal growth factor; 5 μg ml^−1^ insulin; 20 mM D-glucose; 2% fetal calf serum; and 20 mM Hepes, pH 7.4) at 37 °C in 5% CO_2_/95% air. Except for intracellular calcium assay, the mpkCCD cells were seeded on semipermeable filters (Transwell, 0.4-μm pore size; Corning Costar), and the medium was changed every day. For immunocytochemistry, 0.33-cm^2^ filters were used, and for western blot and PCR analysis, 4.67-cm^2^ filters were used. The mpkCCD cells were cultured for 5 days and were then serum-starved and hormone-deprived for 12 h to reduce the basal activity of Wnt signalling. Wnt5a (R&D Systems), [deamino-Cys1, d-Arg8]-vasopressin (dDAVP) (Sigma-Aldrich), arachidonic acid (Sigma-Aldrich), N-(6-aminohexyl)-5-chloro-1-naphthalenesulfonamide (W7) (Sigma-Aldrich), cyclosporin A (CyA) (Wako), KN93 (Sigma-Aldrich), H89 (Sigma-Aldrich), and Y27632 (Nacalai Tesque) were applied to the basolateral side of the mpkCCD cells. Indomethacin (Sigma-Aldrich) was applied to both sides of the mpkCCD cells. In intracellular calcium assay, the mpkCCD cells were seeded on 35-mm μ-Dish culture plates (Ibidi). The mpkCCD cells were cultured for 24 h and were then serum-starved and hormone-deprived for 12 h.

### Animal experiments

Animal studies were performed using 8-week-old C57BL/6 male mice (CLEA JAPAN) that had free access to food and water. The mice were subcutaneously injected with tolvaptan (LKT Laboratories) or the same amount of DMSO (Sigma-Aldrich) control solution for 2 days by osmotic minipumps (Alzet model 1003D) as previously described^42^. The mice were then intraperitoneally injected with 300 μl of Wnt5a or PBS control. Urine samples were collected within 2 h after the administration of Wnt5a. Urine osmolality was measured with a Fiske One-ten Osmometer. Tissue samples for western blot and immunofluorescence were obtained 1 h after the administration of Wnt5a. The animal experiments were approved by The Animal Care and Use Committee of Tokyo Medical and Dental University.

### Western blot analysis

Whole homogenates of mouse kidneys without the nuclear fraction (600 × *g*) were prepared, and the crude membrane fraction (17,000 × *g*) was used to measure the levels of AQP2 as previously described^56^. The mpkCCD cells were washed twice with phosphate-buffered saline (PBS) and were then solubilized in 200 μl of lysis buffer (50 mM Tris/HCl, pH 7.5, 150 mM NaCl, 1 mM EGTA, 1 mM EDTA, 50 mM sodium fluoride, 1 mM sodium orthovanadate, 1% Triton X-100, 0.27 M sucrose, 1 mM DTT and Complete protease inhibitor cocktail (Roche) (1 tablet per 50 ml)) as previously described^57^. After centrifugation at 15,000 × *g* for 10 min at 4 °C, the protein concentration was measured using the Bradford (Expedeon) protein assay method. The supernatants were denatured in SDS sample-buffer (Cosmo Bio) for 30 min at 37 °C. Equal amounts of protein were separated by SDS–PAGE and were transferred to nitrocellulose membrane (GE Healthcare Life Sciences). The blots were probed with the following primary antibodies: goat anti-AQP2 (N-20, Santa Cruz, sc-9880; 1:1,000), rabbit anti-AQP2 (phospho S256, Abcam, ab109926; 1:1,000), rabbit anti-AQP2 (phospho S261, Symansis, p112-261; 1:1,000), rabbit anti-AQP2 (phospho S269, Symansis, p112-269; 1:1,000), mouse anti-β-actin (Sigma-Aldrich, A2228; 1:1,000), rabbit anti-phospho-PKA substrate (Cell Signaling, #9624; 1:1,000), mouse anti-Dvl2 (Santa Cruz, sc-8026; 1:1,000), rabbit anti-Dvl3 (Cell Signaling, #3218; 1:1,000), rabbit anti-β-catenin (Cell Signaling, #8480; 1:1,000), rabbit anti-phospho-β-catenin (Ser675) (Cell Signaling, #4176; 1:1,000), mouse anti-Flag (Sigma-Aldrich, F3165; 1:1,000), rabbit anti-Frizzled 2 (abcam, ab52565; 1:250), goat anti-Frizzled 4 (C-18, Santa Cruz, sc-66450; 1:200), rabbit anti-Frizzled 5 (Millipore, 06-756; 1:1,000), and goat anti-Frizzled 6 (E-19, Santa Cruz, sc-32148; 1:200). Alkaline phosphatase-conjugated anti-IgG antibody (Promega) was used as the secondary antibody, and Western Blue (Promega) was used to detect the signals. The band intensities of the Western blots were quantified using ImageJ software.

### Immunofluorescence studies

Mouse kidneys were fixed by perfusion through the left ventricle with 0.2 M periodate lysine and 2% paraformaldehyde in PBS. Tissue samples were soaked for several hours in 20% sucrose in PBS, embedded in Tissue-Tek OCT Compound (Sakura Finetechnical), and snap-frozen in liquid nitrogen. Goat anti-AQP2 (C-17, Santa Cruz, sc-9882; 1:500) was used as the primary antibody. The mpkCCD cells were fixed with 4% paraformaldehyde and permeabilized with 0.1% Triton-X/PBS. The filters were detached from the holders and incubated with the following primary antibodies: goat anti-AQP2 (N-20; 1:500), rabbit anti-AQP2 (phospho S269; 1:500), and Acti-stain 555 phalloidin (Cytoskeleton, # PHDH1-A; 7:1,000). Alexa 488 and 546 dye-labeled antibodies (Molecular Probes; 1:200) were used as the secondary antibodies. The samples were mounted with Vectashield/DAPI (Vector Laboratories). Immunofluorescence images were obtained using the LSM510 Meta (Carl Zeiss). The fluorescence intensities of AQP2 in mouse kidneys were quantified using Zen 2009 software. The fluorescence intensities of AQP2 in the mpkCCD cells were quantified using ImageJ software.

### Reverse transcription-PCR analysis

Total RNA from the mpkCCD cells and C57BL/6 mouse kidneys was extracted using TRIzol Reagent (Invitrogen) and purified with RNase-free DNase Sets and RNeasy Kits (Qiagen). RNAs were reverse-transcribed using Omniscript Reverse Transcriptase (Qiagen). Primer sequences used for PCR were summarized in Supplementary Table 1.

### Quantitative real-time PCR analysis

Total RNA was extracted using TRIzol Reagent (Invitrogen) and was reverse-transcribed using Omniscript Reverse Transcriptase (Qiagen). Quantitative real-time PCR analysis was performed in a Thermal Cycler Dice Real Time System (Takara Bio). Primers and templates were mixed with SYBR Premix Ex Taq II (Takara Bio). Transcript levels were normalized to GAPDH mRNA levels, and the amount of RNA was calculated using the comparative C_T_ method. Primers used for detection of mouse *AQP2* were 5′-GCC CTG CTC TCT CCA TTG-3′ and 5′-GTT GTC ACT GGC AAG TTT GA-3′. The primer set for *GAPDH* was purchased from Takara Bio.

### Generation of plasmid vectors

To generate p3 × FLAG-CMV10-mFzd receptors, the mouse *Fzd2*, *Fzd4*, *Fzd5* and *Fzd6* coding regions were amplified from mpkCCD-derived cDNA by PCR and were cloned into p3 × FLAG-CMV10 using the Gibson assembly technique (New England Biolabs).

### Intracellular calcium assay

The culture medium of the mpkCCD cells or dissection solution of mouse kidneys was replaced with loading buffer containing 5 μg ml^−1^ Fluo4-AM (Dojindo), 1.25 mmol l^−1^ probenecid (Dojindo), and 0.04% Pluronic F-127 (Dojindo). After incubation for 1 h at 37 °C, loading buffer was replaced with recording medium containing 1.25 mmol l^−1^ probenecid. The mpkCCD cells and isolated CCD of mouse kidneys were then placed under confocal laser scanning microscopy. Wnt5a (500 ng ml^−1^) was added, and calcium responses were monitored by the LSM510 Meta. Fluorescence intensities of Fluo4 were quantified from six regions of interest using Zen 2009 software.

### Cell surface biotinylation assay

The amount of AQP2 in the apical plasma membrane was quantitated by apical surface biotinylation as previously described^58^.

### cAMP assay

Intracellular cAMP levels were measured with the BioTrak EIA system (GE Healthcare Life Science) as previously described^14^. dDAVP was used as the positive control. Phosphodiesterase inhibitors were not added to the mpkCCD cells.

### PKA kinase assay

PKA activity was measured using the enzyme-linked immunosorbent assay (ELISA) kit (Enzo Life Sciences), according to the manufacturer's instructions.

### Isolated tubule microperfusion experiments

CCD tubules dissected from 10-week-old C57BL/6 female mice were microperfused *in vitro*. The dissection, perfusion, and bathing solutions were prepared as previously described^59^. CCD tubules were dissected in cooled (10 °C) dissection solution. Tubules were then transferred to a 1.5 ml bathing chamber and microperfused for 1 h before measurements of *P*_f_. The perfusion and bathing solutions were bubbled with 95% O_2_–5% CO_2_ at room temperature. The perfusion solution contained a volume marker, 0.5 mM fluorescein isothiocyanate-dextran (10,000 MW, Molecular Probes) as previously described^60^. The bathing solution was exchanged every 30 min to keep the osmolality. The perfusion rates were 5–10 nl min^−1^. The tubule lengths were 394±87 μm (*n*=6). *P*_f_ was calculated as previously described^59^.

### Statistics

Statistical significance was evaluated by a one-way ANOVA test with multiple comparisons using Tukey's correction. Data are presented as means±s.d. In microperfusion analysis, student's *t*-tests were performed to assess the statistical significance. Data are presented as means±s.e. *P* values <0.05 were considered statistically significant.

### Data availability

The data that support the findings of this study are available from the corresponding author upon reasonable request.

## Additional information

**How to cite this article:** Ando, F. *et al*. Wnt5a induces renal AQP2 expression by activating calcineurin signalling pathway. *Nat. Commun.* **7,** 13636 doi: 10.1038/ncomms13636 (2016).

**Publisher's note**: Springer Nature remains neutral with regard to jurisdictional claims in published maps and institutional affiliations.

## Supplementary Material

Supplementary Information Supplementary Figures 1-12 and Supplementary Table 1.
