# Supplementary material for: Wnt5a induces renal AQP2 expression by activating calcineurin signalling pathway
Source: Nat Commun. 2016 Nov 28;7:13636. doi: 10.1038/ncomms13636 (PMC5133730; doi:10.1038/ncomms13636)
Supplement: Supplementary Information — Supplementary Figures 1-12 and Supplementary Table 1. [file ncomms13636-s1.pdf]

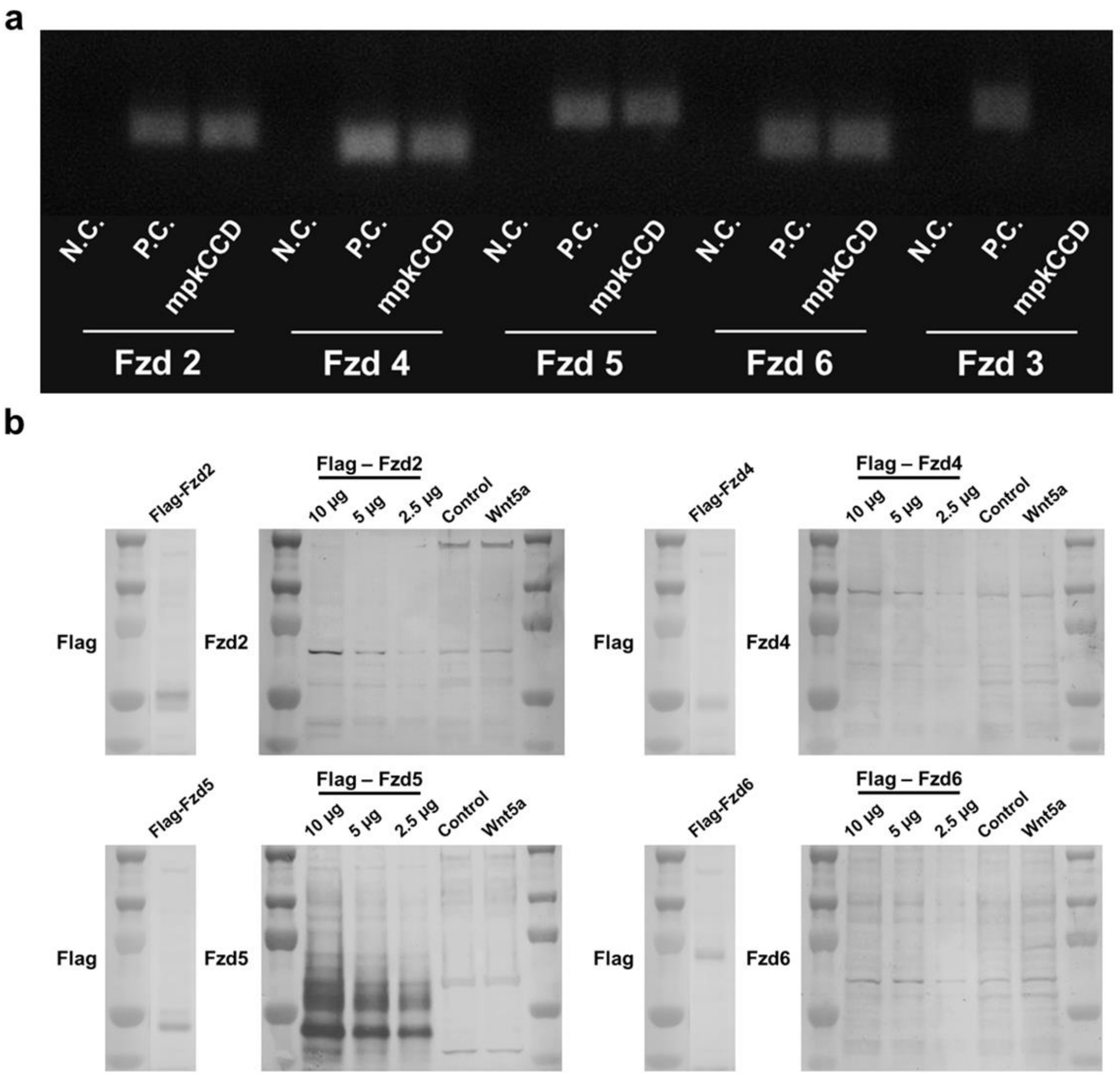

Supplementary Fig. 1. Endogenous Fzd receptors are expressed in the mpkCCD cells. (**a**) mRNA expression of Fzd receptors. mRNA was extracted from the mpkCCD cells and was treated with DNase before RT-PCR assessment. N.C. indicates negative control (water in place of cDNA); P.C. indicates positive control (C57BL/6 mouse kidney). (**b**) Protein expression of Fzd receptors. Flag-Fzd 2,4,5, and 6 were overexpressed in the mpkCCD cells. Anti-Flag antibody detects bands of Flag-Fzd receptors, but commercially available anti-Fzd receptor antibodies except Fzd5 could not detect the overexpressed proteins.

Endogenous Fzd5 in the mpkCCD cells was also not detected with or without Wnt5a (500 ng/ml) treatment for 1h.

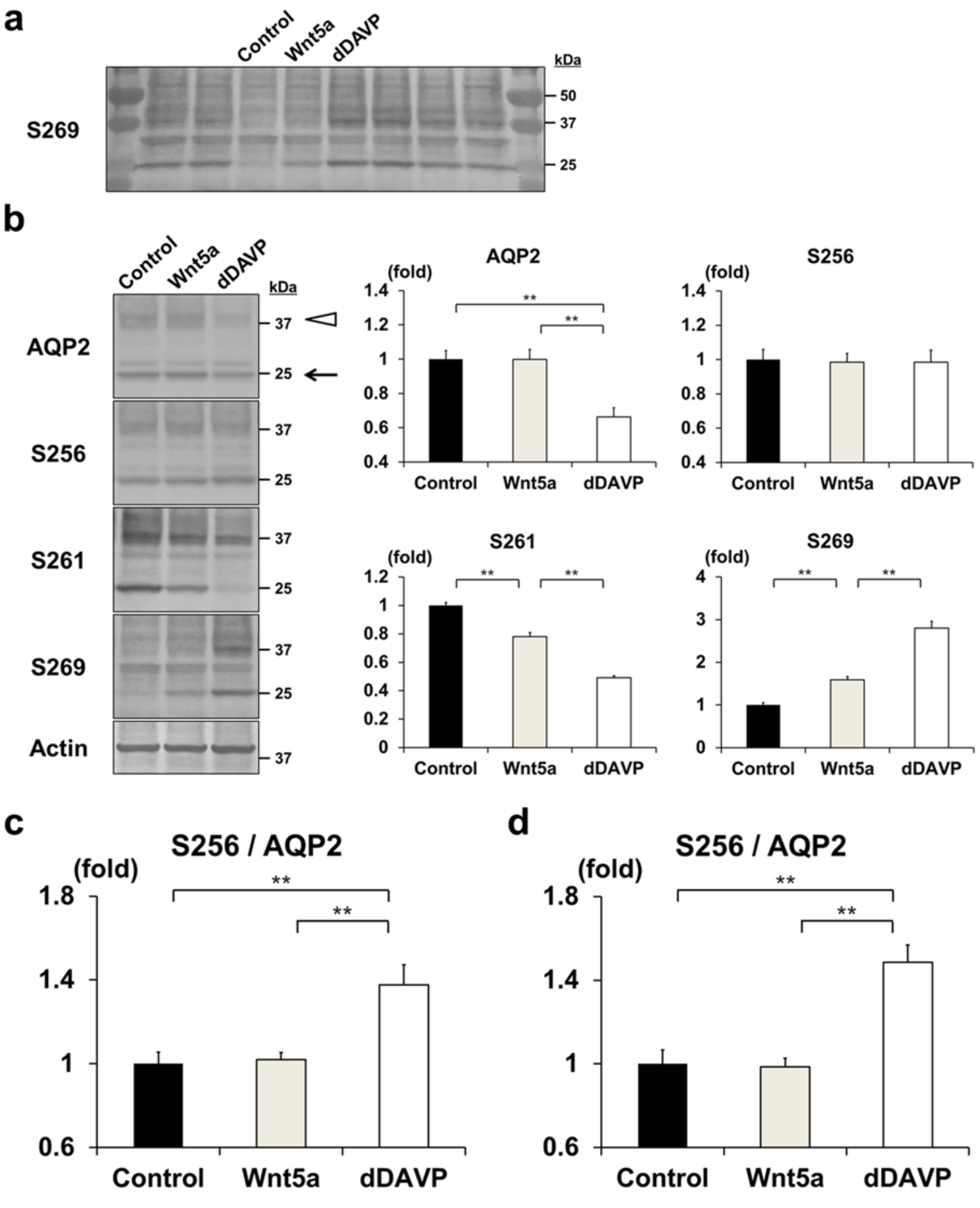

Supplementary Fig. 2. Wnt5a alters AQP2 phosphorylation at S261 and S269. (**a**) Uncropped Western blot of phosphorylated AQP2 at S269. Molecular weight markers (kDa) are shown on the right. (**b**) Densitometric analysis of Figure 1a.

Glycosylated AQP2 bands (arrowhead) were quantified by densitometric analysis, and the results are presented in the bar graphs as fold change compared with the value in the control cells. Bars are mean values ± SD from three experiments. Tukey, $^{**}P < 0.01$. (**c,d**) No significant phosphorylation of AQP2 at S256 in response to Wnt5a. The ratio of phosphorylated AQP2 at S256 to total AQP2 is shown. **c** indicates the ratio of non-glycosylated (arrow) AQP2 bands, and **d** indicates the ratio of glycosylated (arrowhead) AQP2 bands. Tukey, $^{**}P < 0.01$.

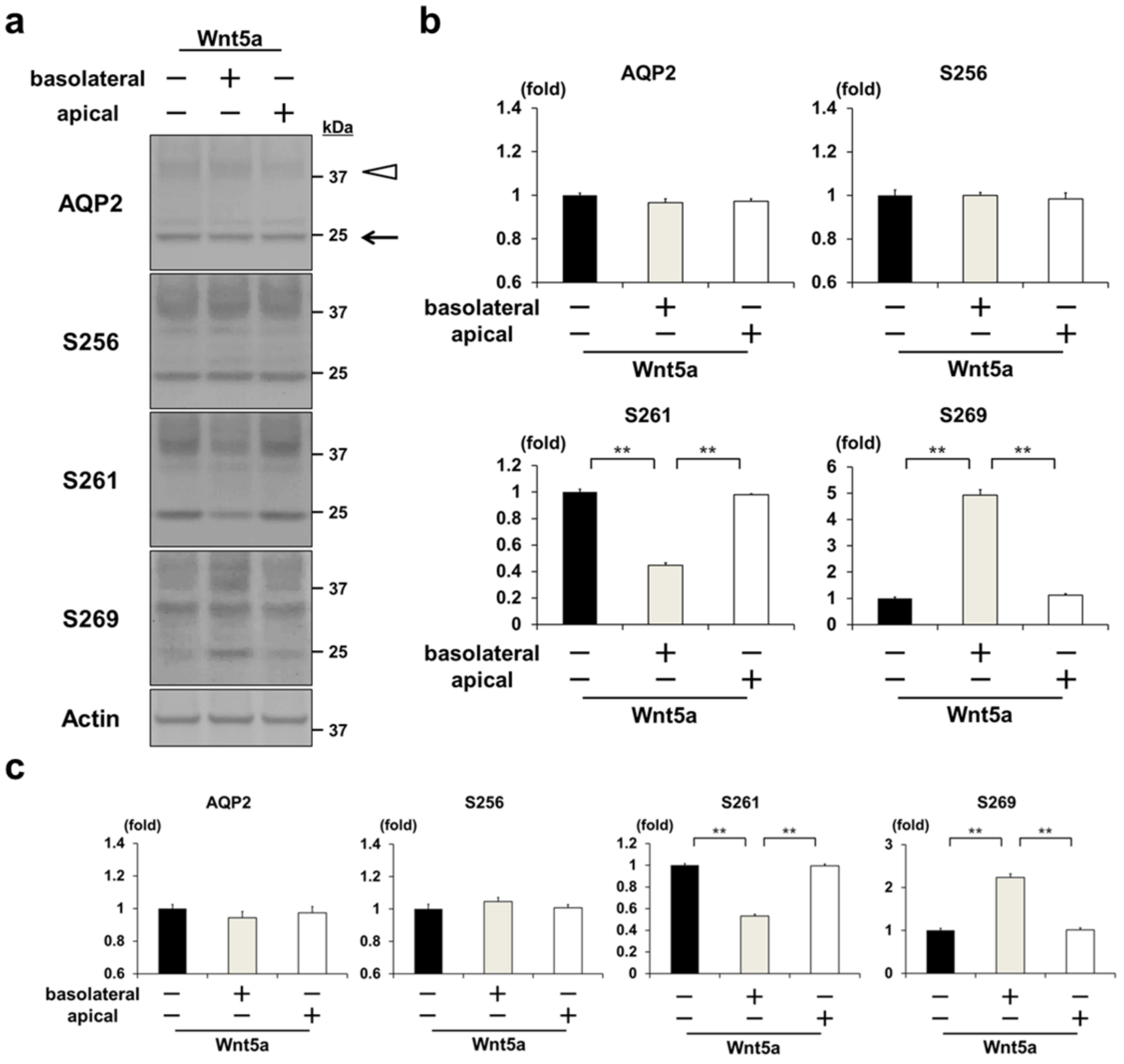

Supplementary Fig. 3. Administration of Wnt5a from apical side of the mpkCCD cells does not change AQP2 phosphorylation. (**a**) Western blot analysis of total and phosphorylated AQP2. Wnt5a (500 ng/ml) was added to the apical or basolateral side of the mpkCCD cells for 1h. (**b**) Densitometric analysis of non-glycosylated AQP2 bands (arrow). The results are presented in the bar graphs as fold change compared with the value in the

control cells. Bars are mean values ± SD from three experiments. Tukey, **$P < 0.01$. (**c**) Densitometric analysis of glycosylated AQP2 bands (arrowhead). The results were analyzed as in **b**. Bars are mean values ± SD from three experiments. Tukey, **$P < 0.01$.

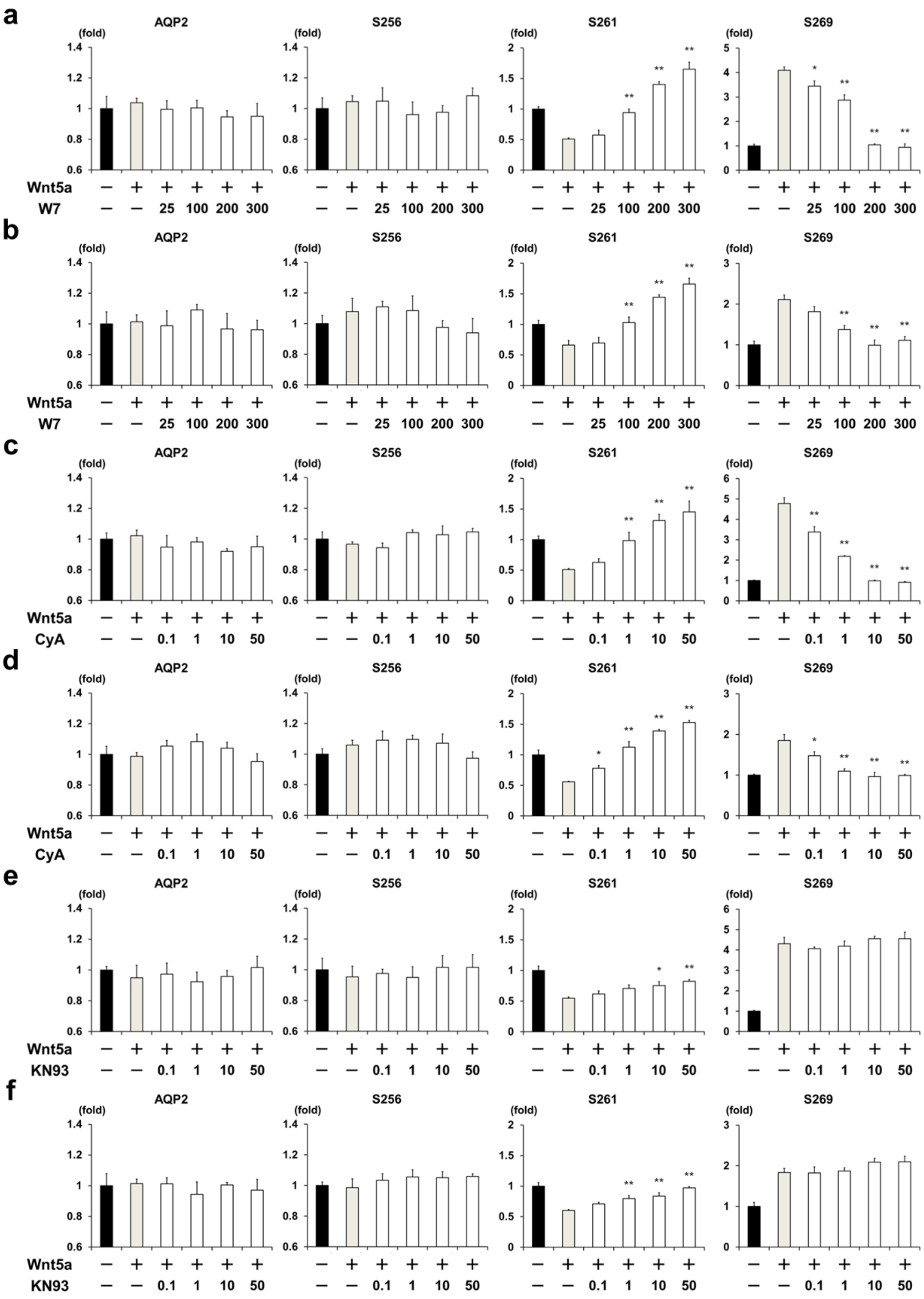
a
AQP2
S256
S261
S269
(fold)
Wnt5a
W7
— — 25 100 200 300
b
AQP2
S256
S261
S269
(fold)
Wnt5a
W7
— — 25 100 200 300
c
AQP2
S256
S261
S269
(fold)
Wnt5a
CyA
— — 0.1 1 10 50
d
AQP2
S256
S261
S269
(fold)
Wnt5a
CyA
— — 0.1 1 10 50
e
AQP2
S256
S261
S269
(fold)
Wnt5a
KN93
— — 0.1 1 10 50
f
AQP2
S256
S261
S269
(fold)
Wnt5a
KN93
— — 0.1 1 10 50

Supplementary Fig. 4. W7 and CyA inhibit Wnt5a-induced AQP2 phosphorylation at S261 and S269. (**a-f**) Densitometric analysis of Figure 1c. AQP2 bands were quantified by densitometric analysis, and the results are presented in the bar graphs as fold change compared with the value in the control cells. **a,c,e** indicate the densitometric analysis of non-glycosylated AQP2 bands (arrow), and **b,d,f** indicate the densitometric analysis of glycosylated AQP2 bands (arrowhead). Bars are mean values ± SD from three experiments. Asterisk indicates a significant difference compared with wnt5a samples. Tukey, $*P < 0.05$, $**P < 0.01$.

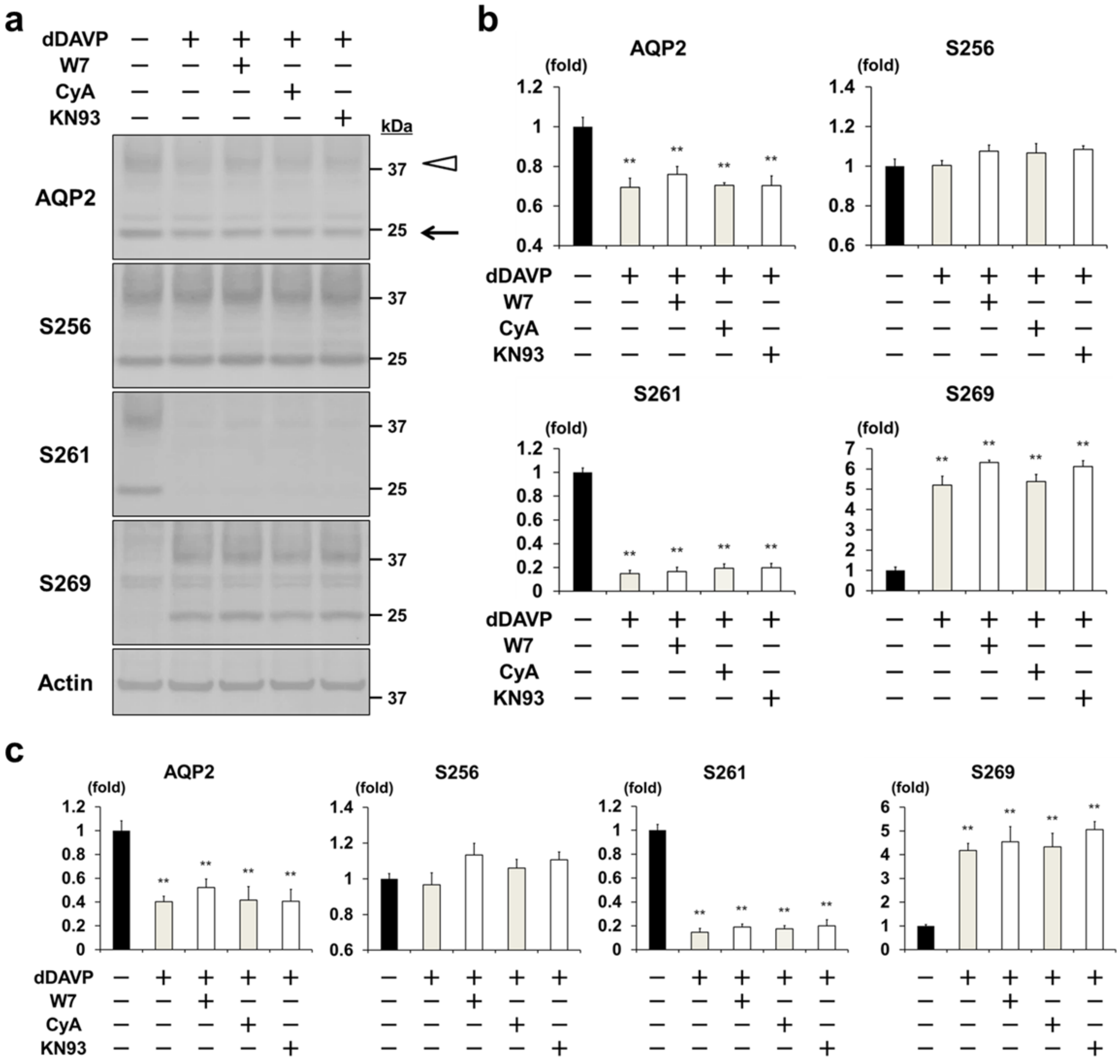

Supplementary Fig. 5. W7, CyA, and KN93 do not affect dDAVP-induced AQP2 phosphorylation. (**a**) Western blot analysis of total and phosphorylated AQP2. dDAVP (1 nM) was added in the presence or absence of W7 (100 µM), CyA (10 µM), or KN93 (10 µM) for 1 h. The mpkCCD cells were pretreated with each inhibitor for 45 min before dDAVP stimulation. (**b**) Densitometric analysis of non-glycosylated AQP2 bands (arrow).

The results are presented in the bar graphs as fold change compared with the value in the control cells. Bars are mean values ± SD from three experiments. Asterisk indicates a significant difference compared with control. Tukey, $**P < 0.01$. (**c**) Densitometric analysis of glycosylated AQP2 bands (arrowhead). The results were analyzed as in **b**. Bars are mean values ± SD from three experiments. Asterisk indicates a significant difference compared with control. Tukey, $**P < 0.01$.

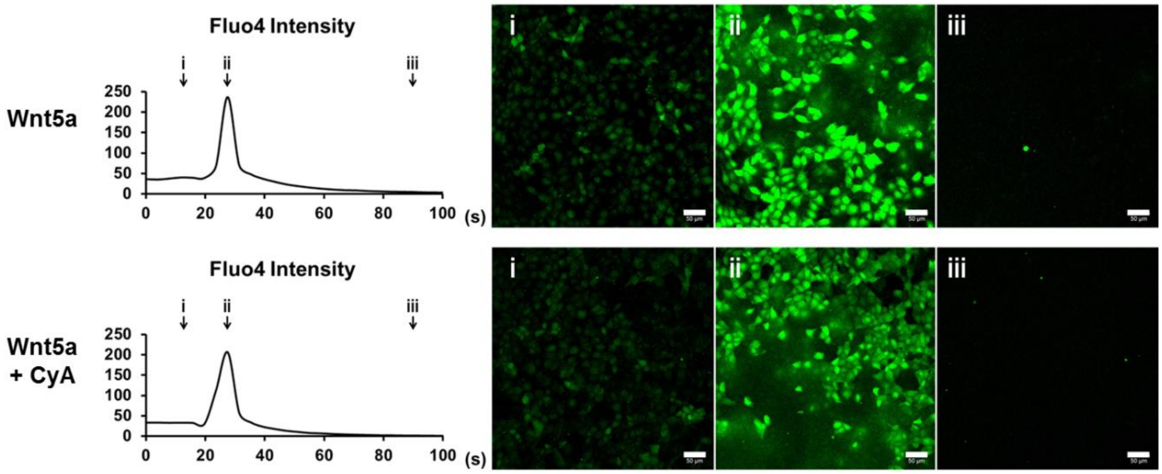

Supplementary Fig. 6. CyA does not inhibit the increase of Fluo4 intensity by Wnt5a. (Left) Wnt5a (500 ng/ml) was added in the presence or absence of CyA (10 μM). The mpkCCD cells were pretreated with CyA for 45 min before Wnt5a stimulation. The time-course of Fluo4 fluorescence intensities are shown. The x axis indicates time, and the y axis indicates Fluo4 intensity. (Right) Representative confocal images at the indicated time (arrow) are shown. Scale bars, 50 μm.

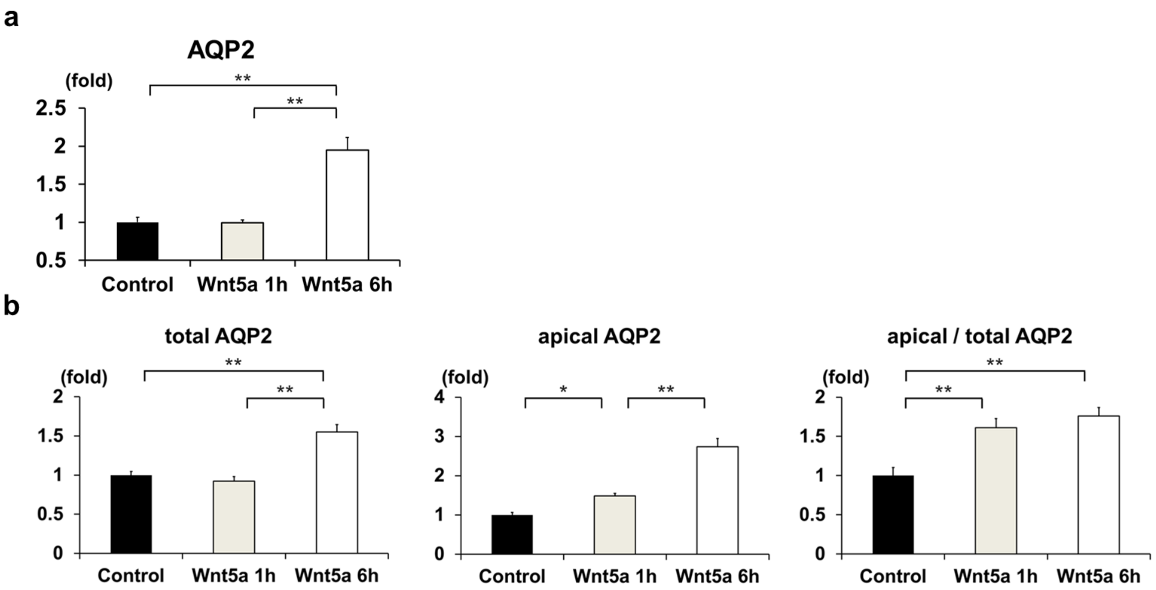

Supplementary Fig. 7. Wnt5a increases AQP2 protein expression and apical AQP2 expression. (**a**) Densitometric analysis of Figure 3d. Glycosylated AQP2 bands (arrowhead) were quantified by densitometric analysis, and the results are presented in the bar graphs as fold change compared with the value in the control cells. Bars are mean values ± SD from three experiments. Tukey, $^{**}P < 0.01$. (**b**) Densitometric analysis of Figure 3e. (Left) Non-glycosylated total and apical (biotinylated) AQP2 bands (arrow) were quantified by densitometric analysis, and the results are presented in the bar graphs as fold change compared with the value in the control cells. Bars are mean values ± SD from three experiments. (Right) The ratio of apical AQP2 to total AQP2 is shown. Tukey, $^{*}P < 0.05$, $^{**}P < 0.01$.

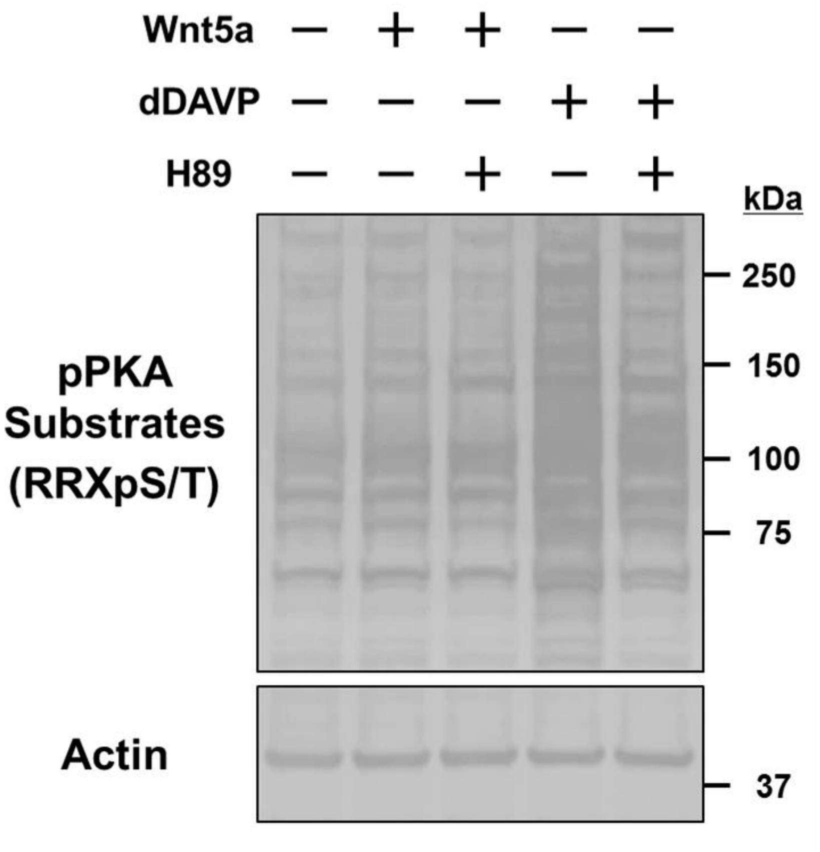

Supplementary Fig. 8. Wnt5a does not increase phosphorylation of PKA substrates. Wnt5a (500 ng/ml) or dDAVP (1nM) was added in the presence or absence of H89 (50 μM) for 1 h. The mpkCCD cells were pretreated with H89 for 45 min before Wnt5a or dDAVP stimulation.

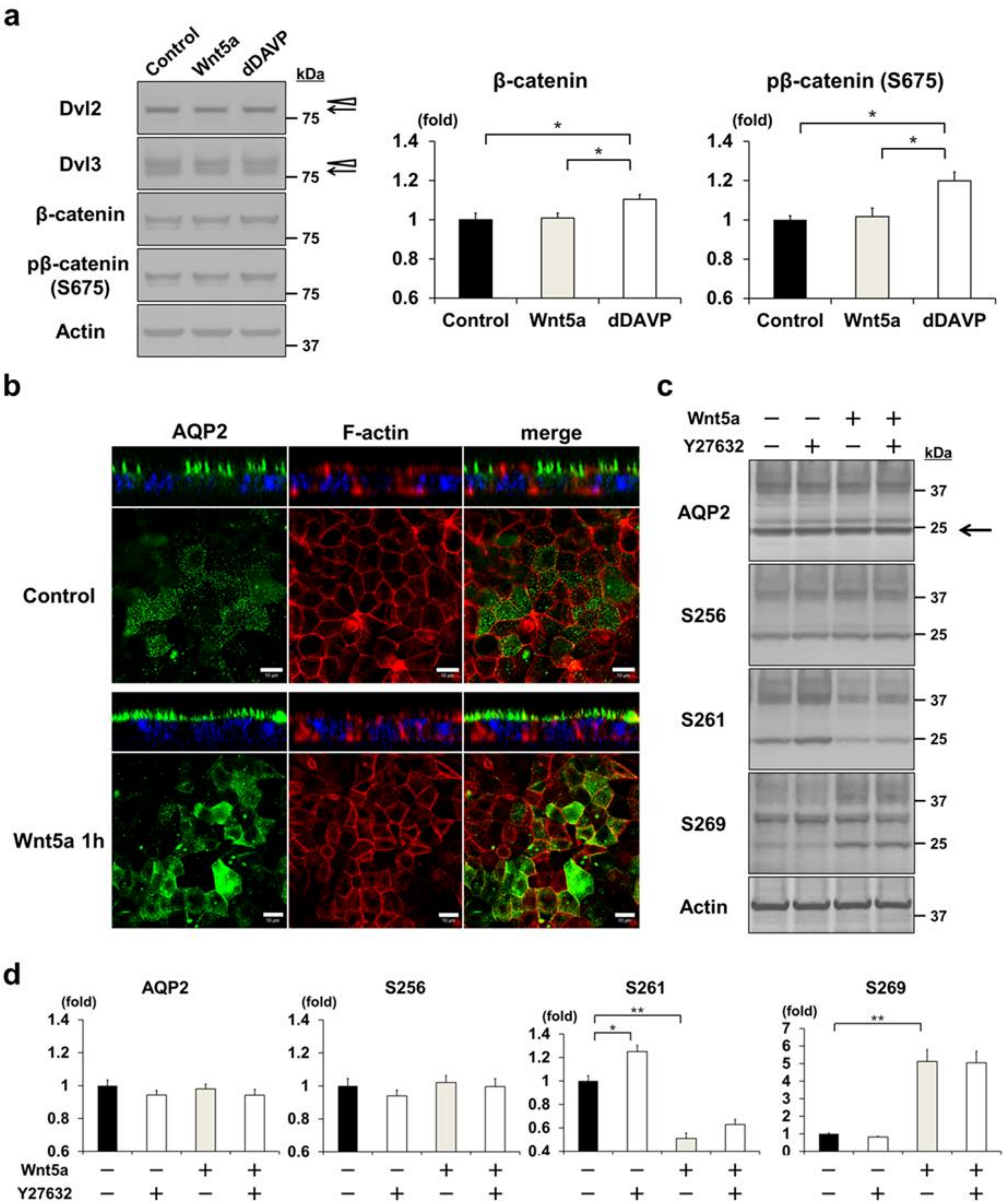

a
Control
Wnt5a
dDAVP
kDa
Dvl2
Dvl3
β-catenin
pβ-catenin (S675)
Actin
75
37
β-catenin
pβ-catenin (S675)
(fold)
1.4
1.2
1
0.8
0.6
Control
Wnt5a
dDAVP
b
AQP2
F-actin
merge
Control
Wnt5a 1h
c
Wnt5a
Y27632
kDa
AQP2
S256
S261
S269
Actin
37
25
d
AQP2
S256
S261
S269
(fold)
Wnt5a
Y27632

Supplementary Fig. 9. Wnt5a does not affect Dvl, β-catenin, and Rho kinase. (**a**) Western blot analysis of Dvl and β-catenin. (Left) Wnt5a (500 ng/ml) or dDAVP (1 nM) was added to the mpkCCD cells for 1h. The position of dephosphorylated (arrow) and phosphorylated (arrowhead) Dvl are indicated. Representative blots of three independent experiments are shown. (Right) Total and phosphorylated β-catenin bands were quantified by densitometric analysis, and the results are presented in the bar graphs as fold change compared with the value in the control cells. Bars are mean values ± SD from three experiments. Tukey, *P < 0.05. (**b**) The effect of Wnt5a on F-actin. Wnt5a (500 ng/ml) was added to the mpkCCD cells for 1h. The larger panels display confocal sections of the apical regions of the cells. Z-stack confocal images are shown at the top of each panel. Representative confocal images of three independent experiments are shown. Scale bars, 10 µm. (**c,d**) The effects of Y27632 on AQP2 phosphorylation. Wnt5a (500 ng/ml) was added in the presence or absence of Y27632 (10µM) for 1 h. The mpkCCD cells were pretreated with Y27632 for 45 min before Wnt5a stimulation. Non-glycosylated AQP2 bands (arrow) were quantified by densitometric analysis, and the results are presented in the bar graphs as fold change compared with the value in the control cells. Bars are mean values ± SD from three experiments. Tukey, *P < 0.05. **P < 0.01.

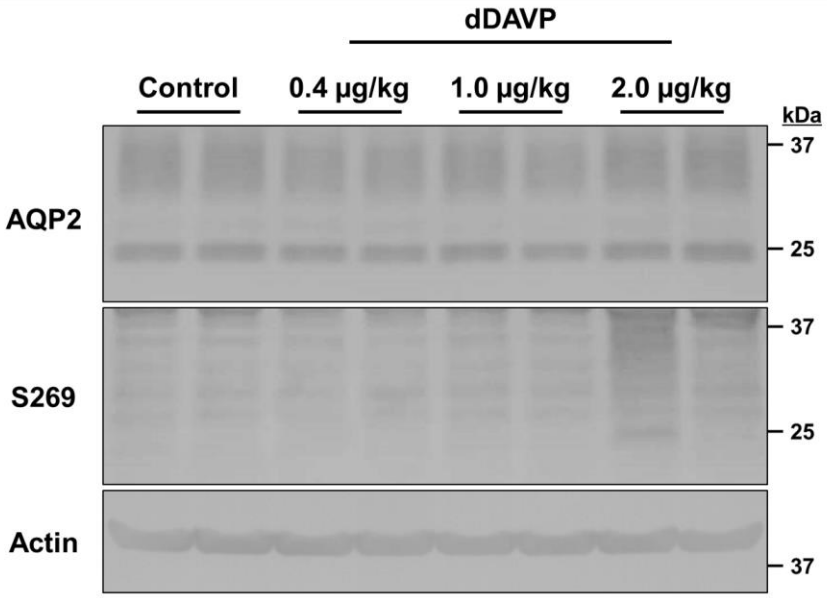

Supplementary Fig. 10. High-dose dDAVP is required to detect pS269-AQP2 bands. Western blot analysis of the membrane fraction of AQP2 in mouse kidneys. The C57BL/6 mice were intraperitoneally injected with dDAVP (0.4, 1.0, 2.0 µg/kg) or distilled water for 1h.

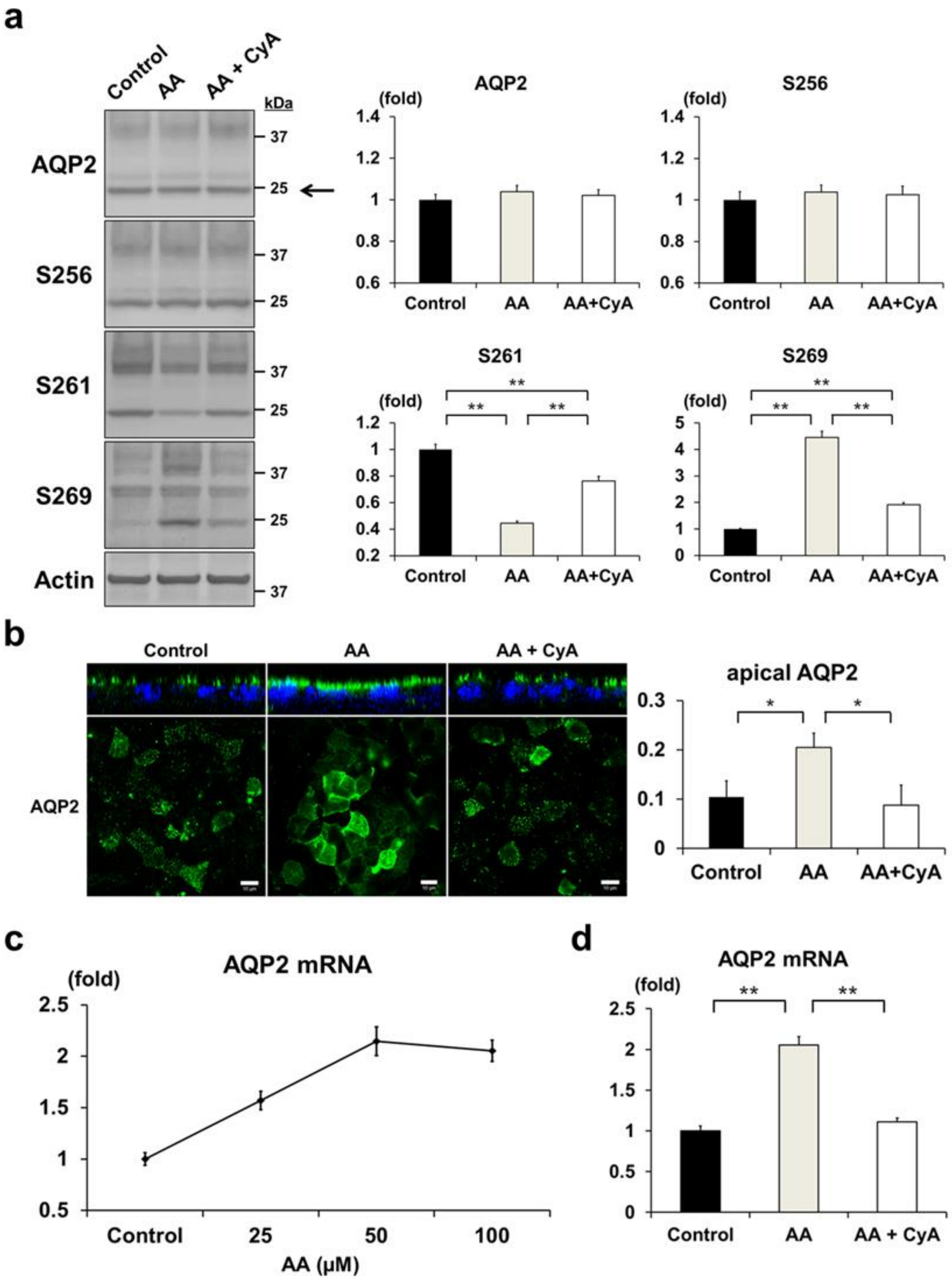

Supplementary Fig. 11. AA regulates AQP2 phosphorylation, trafficking, and mRNA expression. (**a**) Western blot analysis of total and phosphorylated AQP2. (Left) AA (50

µM) was added in the presence or absence of CyA (10µM) for 1 h. The mpkCCD cells were pretreated with CyA for 45 min before AA stimulation. (Right) Non-glycosylated AQP2 bands (arrow) were quantified by densitometric analysis, and the results are presented in the bar graphs as fold change compared with the value in the control cells. Bars are mean values ± SD from three experiments. Tukey, $^{**}P < 0.01$. (**b**) The inhibitory effect of CyA on AA-induced AQP2 trafficking. (Left) AA (50 µM) was added in the presence or absence of CyA (10µM) for 1 h. The mpkCCD cells were pretreated with CyA for 45 min before AA stimulation. The larger panels display confocal sections of the apical regions of the cells. Z-stack confocal images are shown at the top of each panel. Scale bars, 10 µm. (Right) Fluorescence intensities of apical AQP2 were quantified, and the results are presented in the bar graphs. Bars are mean values ± SD from three experiments. Tukey, $^{*}P < 0.05$. (**c**) Dose-response curve of AQP2 mRNA expression in response to AA. The mpkCCD cells were treated with the indicated concentrations of AA for 4 h. AQP2 mRNA expression was examined by quantitative real-time PCR. Results are presented as fold change compared with the value in the control cells. The x axis indicates the concentration of the AA, and the y axis indicates the relative fold change of the AQP2 mRNA. Each value is presented as mean ± SD from three experiments. (**d**) Inhibition of AA-induced AQP2 mRNA expression by CyA. AA (50 µM) was added in the presence or absence of CyA (10µM) for 4 h. The mpkCCD cells were pretreated with CyA for 45 min before AA stimulation. AQP2 mRNA expression was examined by quantitative real-time PCR. Results are presented as fold change compared with the value in the control cells. Bars are mean values ± SD from three experiments. Tukey, $^{**}P < 0.01$.

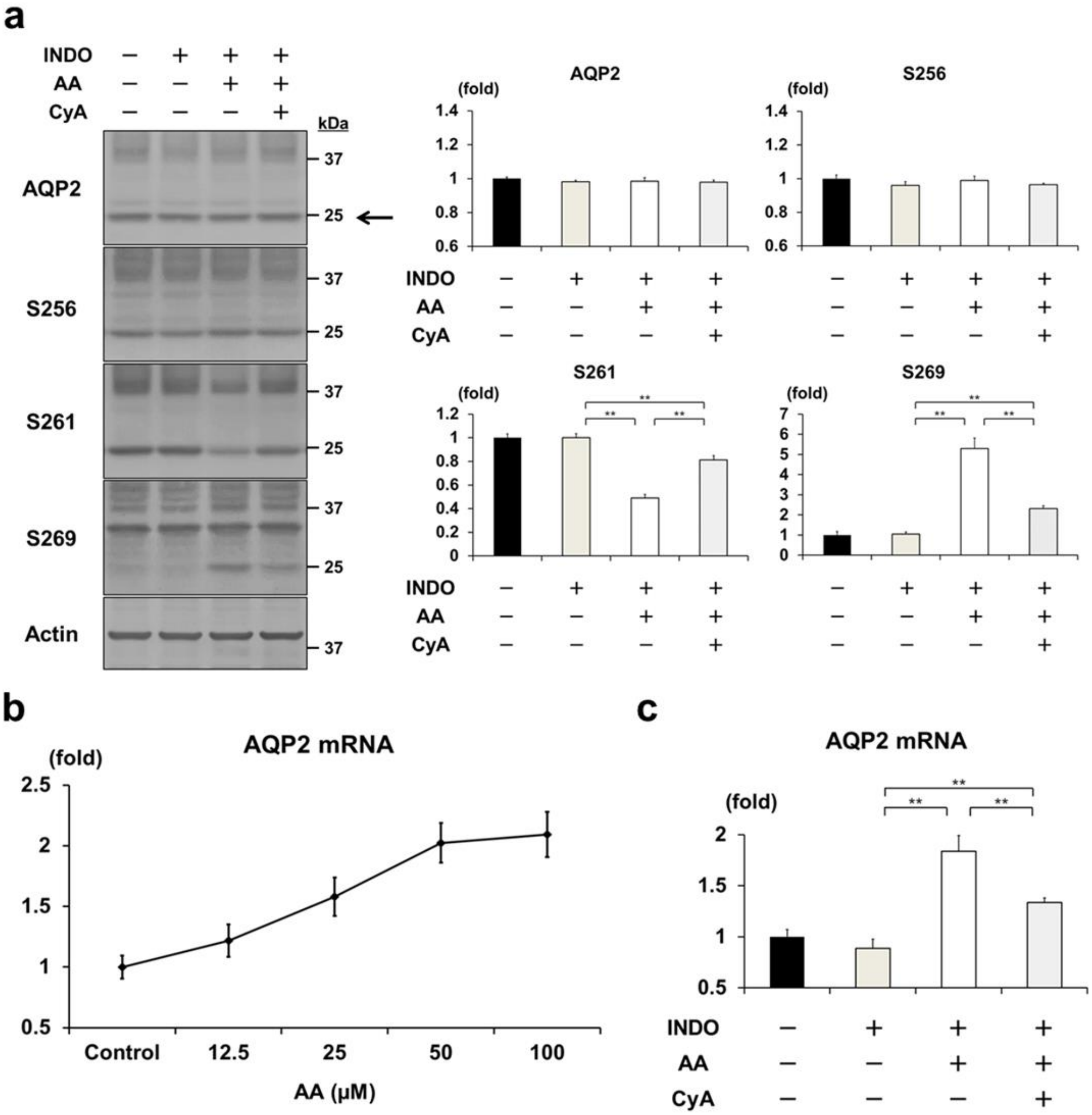

Supplementary Fig. 12. Indomethacin does not affect AA-induced AQP2 phosphorylation and mRNA expression. (**a**) Western blot analysis of total and phosphorylated AQP2. (Left) AA (50 μM) was added in the presence or absence of indomethacin (10 μM) or CyA (10 μM) for 1 h. The mpkCCD cells were pretreated with indomethacin for 36 h or CyA for 45

min before AA stimulation. (Right) Non-glycosylated AQP2 bands (arrow) were quantified by densitometric analysis, and the results are presented in the bar graphs as fold change compared with the value in the control cells. Bars are mean values ± SD from three experiments. Tukey, ***P* < 0.01. (**b**) Dose-response curve of AQP2 mRNA expression in response to AA. The mpkCCD cells were treated with indicated concentrations of AA and indomethacin (10μM) for 4 h after pretreatment with indomethacin for 36 h. AQP2 mRNA expression was examined by quantitative real-time PCR. Results are presented as fold change compared with the value in the control cells. The x axis indicates the concentration of the AA, and the y axis indicates the relative fold change of the AQP2 mRNA. Each value is presented as mean ± SD from three experiments. (**c**) Inhibition of AA-induced AQP2 mRNA expression by CyA. AA (50 μM) was added in the presence or absence of indomethacin (10μM) or CyA (10μM) for 4 h. The mpkCCD cells were pretreated with indomethacin for 36 h or CyA for 45 min before AA stimulation. AQP2 mRNA expression was examined by quantitative real-time PCR. Results are presented as fold change compared with the value in the control cells. Bars are mean values ± SD from three experiments. Tukey, ***P* < 0.01. INDO indicates indomethacin.

Supplementary Table 1. Primer sequences

Gene	Forward primer (5′→3′)	Reverse primer (5′→3′)
Fzd2	CCG ACG GCT CTA TGT TCT TC	TAG CAG CCG GAC AGA AAG AT
Fzd3	GCT GCA GAG AGT ATC ACA	CGG CTC TCA TTC ACT ATC TC
Fzd4	CTG CAG TTC TTC CTT TGT TC	AAC CCA AAT TCT CTC AGG AC
Fzd5	GAC GCC GAG GTT CTG TGT AT	TGC GCA CCT TGT TGT AGA GT
Fzd6	TGG CCT GAA GAA CTT GAA TG	CTC TTG GGA CTT GAT CTG AT
